# Advances in GLP-1 receptor targeting radiolabeled agent development and prospective of theranostics

**DOI:** 10.7150/thno.38366

**Published:** 2020-01-01

**Authors:** Irina Velikyan, Olof Eriksson

**Affiliations:** 1 Department of Medicinal Chemistry, Uppsala University, Uppsala, Sweden; 2 Science for Life Laboratory, Department of Medicinal Chemistry, Uppsala University, Uppsala, Sweden

**Keywords:** Exendin-4, insulinoma, GLP-1, diabetes, PET, SPECT

## Abstract

In the light of theranostics/radiotheranostics and prospective of personalized medicine in diabetes and oncology, this review presents prior and current advances in the development of radiolabeled imaging and radiotherapeutic exendin-based agents targeting glucagon-like peptide-1 receptor. The review covers chemistry, preclinical, and clinical evaluation. Such critical aspects as structure-activity-relationship, stability, physiological potency, kidney uptake, and dosimetry are discussed.

## Introduction

The pancreatic beta cells are crucial for the body's glucose metabolism. Change in beta cell mass (BCM) is implicated in several disorders, for example decrease in BCM is a hallmark of diabetes, while uncontrolled growth of BCM leads to neuroendocrine cancer 1, 2. The clinical value of targeting GLP-1R has been demonstrated in diabetes with medicinal products used to stimulate insulin release, and in cancer for diagnostic imaging of e.g. insulinoma tumors. This review presents evolution of agents for Positron Emission Tomography (PET) and Single Photon Emission Computed Tomography (SPECT) imaging of GLP-1R. It addresses the key aspects of the agent chemistry and biological function, biodistribution, dosimetry, and feasibility of theranostics and radiotheranostics. The potential of targeting GLP-1R in the context of personalized medicine wherein GLP-1R targeted imaging provides basis for individualized treatment is discussed. GLP-1 analogues meet the prior prerequisite of high receptor binding specificity, but the major challenging aspects such as high sensitivity and resolution required because of the small size of the cells and consequently low amount of GLP-1R, and subtle changes that must be quantified demand more attention.

### Unmet medical needs in diabetes

Diabetes affects hundreds of millions of individuals worldwide and the number is expected to double by year 2035 3-5. Conventional diagnostic plasma markers such as fasting B-glucose, glycated hemoglobin A1c, insulin, C-peptide levels and oral glucose tolerance tests provide important information on beta cell function, but changes in these parameters are not tightly coupled to changes in BCM. In fact, a significant fraction of BCM may be lost already by the time of diagnosis of diabetes using plasma markers 6, 7. These methods do not provide direct and quantitative information of the BCM. Moreover, they are an amalgam of the beta cell response to blood-glucose and downstream metabolic processes and may not always provide a sensitive and reproducible assessment of beta cell function either. Thus, there is an unmet need for early and non-invasive diagnostic tool that would support prevention and treatment of the disease. Moreover, some antidiabetic drugs become ineffective over time and monitoring of the disease status is crucial for the adjustment of the treatment in time. Prediction of the drug efficacy on the individual basis is another value that such method would offer. Islet transplantation is an emerging treatment of type 1 diabetes (T1D) 8, however longitudinal studies are required for the investigation of the islet survival and function during various transplant procedures. Thus, in vivo non-invasive imaging technology such as PET would be of utmost importance for monitoring transplanted beta cells 9, 10.

With a continuously increasing population affected by T1D and type 2 diabetes (T2D) worldwide, there is an unmet clinical need for the treatment and prevention of the disease 11. GLP-1R has been considered a potential target for T2D management since the 1990s. Therapeutic drugs targeting GLP-1R provide glucose control by the incretin effect, i.e. they help the beta cells release increasing amounts of insulin in response to hyperglycemia. Development of novel GLP1R targeting drugs has the potential for improving therapeutic efficacy while reducing side effects (e.g. nausea). However, the drug development process imposes scientific, clinical, and financial challenges. Unfortunately, the failure rate of new drugs, in general, is rather high and it is a costly process. PET offers advantages such as possibility to quantify the target engagement and occupancy very early in the development in vivo in humans due to the microdosing concept 12-14 thus facilitating stratification of candidate drugs. The microdosing concept implies that the drug is administered at sub-therapeutic doses often defined as 1% of the expected therapeutic dose or maximally 100 µg (or 30 nanomoles for macromolecules). Microdosing lessens safety requirements and allows Phase 0 trials, potentially reducing the associated cost substantially. Many GLP-1R agonists are highly potent and may exert pharmacological effects already at dose of 10 µg. In this context, very small doses of radiolabeled compound must be administered to follow the microdosing principles.

The development of novel anti-diabetic therapies targeting GLP-1R would benefit from the employment of PET that would enable in vivo investigation of GLP-1R engagement by the therapeutic agent. Based on such early and safe in vivo drug evaluation in man, it is possible to select or reject the candidate drug optimizing development expenses. Considerable effort is currently directed to the development of dual agonist drugs, e.g. combining activity for both GLP-1 and the glucagon receptor or the gastric inhibitory polypeptide (GIP) receptor 15-18. Such unimolecular drugs with several targets would potentially provide improved glucose control combined with clinically meaningful weight reduction. An early target occupancy investigation using PET in combination with radiolabeled ligands for each of the intended target receptors would play an important role in the acceleration of the development process 19. The level of occupancy of the GLP-1R required for full agonistic effect is not known, but can potentially be studied by PET.

### Unmet medical needs in cancer

Both benign and malignant insulinomas are forms of pancreatic neuroendocrine tumors (PNETs) of beta-cell origin 20-22. In most of the cases (over 90%) they are benign and single, however very difficult to accurately localize using radiological methods such as endosonography, MR and CT prior to surgical excision due to the small size (1-2 cm) 23-28. Moreover, the incomplete resection may cause symptom persistence. They cause hyperinsulinemic hypoglycemia, and although they are rare it is a potentially fatal disease. The density of the somatostatin receptors in benign insulinomas is commonly insufficient for diagnostic imaging, e.g. [^111^In]-pentetreotide (Octreoscan®) failed to detect the small multiple lesions in human examinations. Whereas GLP-1R is expressed with high incidence and density opening the possibility for utilizing exendin-based imaging agents for accurate localization and intraoperative guidance 29-33.

The accuracy of staging is crucial in case of malignant insulinomas, and unfortunately conventional radiological procedures such as magnetic resonance (MR) imaging, endosonography, and Computed Tomography (CT)) are conclusive in <50% 27, 34-36. Selective angiography together with venous sampling for insulin after intra-arterial calcium stimulation administration provides the accuracy of 60-80%, however, it is an invasive procedure with high risk for side effects. Radionuclide-based imaging with metabolic agents such as [^18^F]Fluorodeoxyglucose ([^18^F]FDG)/PET-CT, [^11^C]5-Hydroxytryptophan (5-[^11^C]HTP/PET-CT or [^18^F]DOPA/PET-CT was found insufficiently sensitive 26. In contrast to benign insulinomas, malignant insulinomas express SSTR in density adequate for the imaging, while GLP-1Rs are expressed to much lesser extent or absent 37. Nevertheless, both SPECT and PET clinical studies demonstrated imaging of malignant insulinoma using exendin-4 analogues 26, 38, 39. Targeting both SSTRs and GLP-1Rs could provide complementary diagnostic value wherein negative scan using imaging agents comprising GLP analogues may potentially indicate malignancy 40.

### Targeting glucagon-like peptide 1 receptor (GLP-1R)

Endogenous GLP-1 exists in two forms, GLP-1(7-36)-NH_2_ and GLP-1(7-37)-NH_2_ and belongs to the incretin hormone group responsible for the regulation of blood glucose level. The respective receptor, GLP-1R, is a G-protein coupled receptor of seven-transmembrane topology 23, 32, 33, 41. It is expressed physiologically in pancreas, intestine, lung, kidney, breast and brain, and overexpressed in such pathologies as insulinomas, gastrinomas, and phaeochromocytomas with the highest GLP-1R incidence and density in insulinomas 32, 33, 42. GLP-1R is considerably expressed on the beta cells which constitute approximately 65-80% of the cells in islets of Langerhans, while expression in exocrine pancreas, and other pancreatic endocrine cells (for example, alpha- and delta-cells) has been reported as either absent, low or intermediate in human, depending on study 43-45. GLP1R mRNA transcription in human exocrine pancreas is low. However, some animal models such as pigs seem to exhibit high GLP1R densities in the exocrine pancreas 46. The anti-diabetic function of GLP-1 presents therapeutic interest especially in T2D. Additionally, agonism of the GLP1R has been implicated in promoting beta cell proliferation and regeneration in animal models 47. The major hinder of using endogenous GLP-1 as a drug was its short plasma half-life (< 2 min) 48 and research efforts were directed at the improvement of in vivo stability against dipeptidyl peptidase IV (DPP-IV) and neutral endopeptidase (NEP). It resulted in several anti-diabetic drugs (exenatide/AstraZeneca, liraglutide/Novo Nordisk, taspoglutide/Ipsen-Roche, lixisenatide/Sanofi-Aventis, semaglutide/Novo Nordisk, albiglutide/GalxoSmithKline) based on GLP-1 analogs that stimulate insulin biosynthesis and secretion dependent on the blood glucose level and restoration of beta cell mass and function 18, 49. Research in molecular targeting of GLP-1R expressed on beta cells 43 and involved in various pathological processes, e.g. in insulinomas, gastrinomas, and phaeochromocytomas 23, 32, 33, 41 has been expanding very fast since the development of metabolically stable ligands, e.g. exendin-4. A 39 amino acid residue peptide, exendin-4, isolated from the saliva of Gila monster lizard has plasma half-life of 2.4 h 49, 50. It binds to the same GLP-1R site as GLP-1 does with picomolar activity 51, 52. Exendin-4 has 50% homology with GLP1. Crystal structure studies indicated that exendin-4 forms both hydrophobic and hydrophilic interactions with GLP-1R 52. The pioneer study of biodistribution of radioiodinated exendin-3 (also a GLP-1R agonist, identical to exendin-4 except for two amino acid substitutions) in rat insulinoma model demonstrated the potential of GLP-1R targeted scintigraphy for the insulinoma detection in vivo 53. However, exendin-3 and the radiolabel were not sufficiently stable, and the author warranted further research for the improvement, in particular labeling with radiometals.

### Challenges of in vivo beta cell quantification

It is assumed that the investigation of T1D and T2D pathophysiology mechanism requires distinguishing between BCM and beta cell function 7, 54. Longitudinal invasive tissue sampling of the pancreas to measure BCM by histology is in most circumstances unacceptable due to the complications associated with pancreatic biopsies. Thus, the accurate histochemical determination of the BCM is not normally feasible except in cross-sectional studies using tissue samples from post mortem organ donors. PET and SPECT imaging of beta cells in vivo in humans is challenging since the spatial resolution of the scanners (>4 mm) exceeds at least 10-fold the size of the islets of Langerhans (50-300 µm) comprising beta cells. While this means that it is highly challenging to image individual islets, it is still theoretically and practically feasible to image the beta cell concentration in a given pancreatic volume. Therefore, it is also possible with medical scanners to longitudinally assess the total pancreatic BCM by multiplying the beta cell concentration with the total pancreatic volume. The total mass of beta cells constitutes approximately 2% of the total pancreatic mass and the cells are scattered heterogeneously throughout the pancreatic volume 7, 55. As in similar applications where the target density in tissue is low (e.g. astrocyte imaging in neuroinflammation), high sensitivity of the imaging technology in combination with high specificity of the labeled ligand compensate for the resolution shortcomings. It is suggested to measure the total pancreatic uptake with prerequisite of specific accumulation solely in the beta cells 54, 56. Thus, the prospective of the quantification of the BCM is reduced mainly to the availability of high specificity imaging agents. The extensive investigational work has been conducted preclinically using human tissue bank material, various animal species, and ^68^Ga-, ^111^In- and ^177^Lu-labeled exendin analogues, and the uptake of pancreas was shown to correlated with BCM in murine models 57-59. It is important to stress that as long as GLP-1R is absent on pancreatic acini 57 and islet alpha cells 60 it is feasible to quantify the beta cell uptake of exendin-4 based analogues with high accuracy, despite the limited resolution of PET and SPECT scanners. It is worth mentioning that no correlation of ^177^Lu-labeled exendin-4 uptake and estimated alpha cell mass could be found in a study of transplanted islets in non-diabetic mice 9. The analogues labeled with positron emitting radionuclides potentially provide advantages in terms of higher sensitivity, resolution, accurate quantification, and determination of kinetic parameters describing the uptake mechanism and thus underlying biological processes.

## Imaging agents: chemistry and pre-clinical evaluation

PET and SPECT present strong potential for the in vivo imaging of subnanomolar imaging agent concentration. It has been of strong interest to develop an imaging agent specifically targeted at GLP-1 receptor for non-invasive and quantitative diagnosis. Since the natural agonist, a 30 amino acid residue hormone, is metabolically unstable 61, analogues with prolonged in vivo half-life have been developed 53, 62, 63. A more stable GLP-1R agonist, synthetic exendin-4 (Exenatide) is currently used in diabetic treatment. Molecular imaging agents based on exendin-3 and exendin-4 targeting GLP-1R for SPECT 53, 62-69 and PET 9, 26, 58, 68-87 were developed and demonstrated clinical value of both SPECT 64-66 and PET 26, 59, 75, 87. Various studies have been conducted with the common aim to develop a targeting agent for specific binding to GLP-1R with high affinity. The most challenging aspects that have been addressed are specific radioactivity, structure-activity relationship, in vitro and in vivo stability, high kidney uptake, and high physiological potency of the ligands. A number of imaging agents varying in the peptide sequence, chelator and prosthetic group moiety, and radionuclide (^18^F, ^64^Cu, ^68^Ga, ^111^In, ^99m^Tc, ^177^Lu, ^124/125/131^I, ^89^Zr) has been developed and investigated preclinically and clinically. They demonstrated variability in affinity, pharmacokinetics, and biodistribution. The modulation of kidney uptake was investigated particularly. The respective ligands were labeled with ^111^In 63, 68, 69, ^99m^Tc 65, 69, ^68^Ga 58, 68, 69, 77, 78, and ^64^Cu 71, 77-79, ^18^F 72-74, 80-84, and ^89^Zr 85. The choice of a radionuclide is commonly determined by the purpose of a study and critical characteristics such as availability, decay mode, and labeling chemistry (Table 1).

### Metal radionuclide-based analogues

Exendin-4 was conjugated to either tetraazacyclododecantetraacetic acid (DOTA) or diethylenetriaminepentaacetic acid (DTPA) via aminohexanoic acid linker (Ahx) 62, 63, 69, 88. [Lys^40^(Ahx-DTPA-^111^In) NH_2_]-exendin-4 demonstrated nanomolar affinity and rapid binding and internalization kinetics in INS-1 cells in vitro and high uptake in subcutaneous INS-1 tumors of BALB/c nude mice 67. The uptake of [Lys^40^(Ahx-DTPA-^111^In) NH_2_]-exendin-4 was also detected in GLP-1R positive organs such as stomach, pancreas, lung, adrenals, and pituitary in healthy rats and mice, while no brain accumulation was detected in vivo in mice 62. The agent not only localized tumors in Rip1Tag2 mouse model of pancreatic beta-cell carcinogenesis 63 but also demonstrated radiotherapeutic effect with up to 94% reduction of the tumor volume in a dose dependent fashion and without significant acute organ toxicity 88. The strong potential of [Lys^40^(^68^Ga-DOTA)]-exendin-3 and [Lys^40^(^111^In-DTPA)]-exendin-3 for diagnostic imaging of insulinomas was demonstrated in mice bearing INS-1 xenografts and also for the determination of beta cell mass shown in rat model of alloxan-induced beta cell loss 57, 67, 68. The affinity of [Lys^40^(^111^In -DTPA)]-exendin-3 determined as IC50 in INS cells was in low nanomolar range 67. [Lys^12^(^111^In -BnDTPA-Ahx)]exendin-4 demonstrated specific uptake in mouse pancreatic beta cells and insulinoma xenografts 89, 90.

^99m^Tc presents advantage over ^111^In in lower radiation burden and higher resolution. Exendin-4 labeled with ^99m^Tc ([Lys^40^(Ahx-HYNIC-^99m^Tc/ethylenediaminediacetic acid [EDDA])NH_2_]-exendin-4) demonstrated significantly lower tumor and organ uptake in Rip1Tag2 mouse model of pancreatic beta cell carcinogenesis compared to ^68^Ga and ^111^In labeled counterparts, however the quality and contrast of the image was still sufficiently high 69. The effective dose of [Lys^40^(Ahx-HYNIC-^99m^Tc/EDDA])NH_2_]-exendin-4 was 43 times less than that for [Lys^40^(Ahx-DOTA-^111^In-NH_2_]-exendin-4. Two analogues of [Lys^40^(Ahx-HYNIC-^99m^Tc/EDDA])NH_2_]-exendin-4 containing either methionine or norleucine at position 14 demonstrated similar biodistribution with blockable uptake in GLP-1R positive organs such as lung, pancreas and stomach in normal rats 86. ^99m^Tc-HYNIC-β-Ala-Exedin4 was successfully used to monitor the BCM in mouse model of diet-induced obesity (DIO) and diet-restricted obesity (DRO) wherein DIO considerably reduced the beta cell uptake and DRO failed to normalize the uptake 91. A GMP compliant freeze-dried kit for the preparation of ^99m^Tc-EDDA/HYNIC)-exendin(9-39) was developed and validated for the clinical use in diagnosis of insulinomas 92.

^111^In and ^99m^Tc present such disadvantages as relatively high radiation burden, low spatial resolution and sensitivity as well as poor quantification, that can be overcome by using ^68^Ga. Several analogues based on metabolically stable exendin-3 and exendin-4 have been labeled with ^68^Ga via such chelator moieties as NOTA, NODAGA, DOTA, and DFO conjugated to the peptides at various positions. They were evaluated preclinically in vitro, ex vivo and in vivo for the feasibility of the visualization and quantification of GLP-1R in tumors and pancreatic beta cells, and some of them were compared to their ^111^In- ^99m^Tc, ^64^Cu- and ^86^Zr-labeled counterparts.

[Lys^40^(Ahx-DOTA)NH_2_-exendin-4 labeled with ^68^Ga under microwave heating was biologically evaluated in Rip1Tag2 mouse model of pancreatic beta cell carcinogenesis investigating biodistribution and dosimetry 69. The target localization and blood clearance were fast visualizing as small as 1.5 and 2.3 mm tumors in the mouse pancreas by a human PET/CT scanner. The uptake of 205±59 %ID/g was significantly higher than that of the ^99m^Tc- and ^111^In-counterparts. The effective radiation dose for [Lys^40^(Ahx-DOTA-^68^Ga)NH_2_-exendin-4 was 31.7 µSv/MBq which was 5 times lower and 8 times higher than that for ^111^In- and^ 99m^Tc-counterparts respectively. Exendin-3 labeled with ^68^Ga via DOTA conjugated at Lys^40^ position, [Lys^40^(^68^Ga-DOTA)]-exendin-3, demonstrated somewhat lower uptake in INS-1 xenografts in mice as compared to [Lys^40^(^111^In-DTPA)]-exendin-3^68^. However, given the advantages of PET over SPECT, [Lys^40^(^68^Ga-DOTA)]-exendin-3 was considered as a promising PET imaging agent.

**Figure 1:**
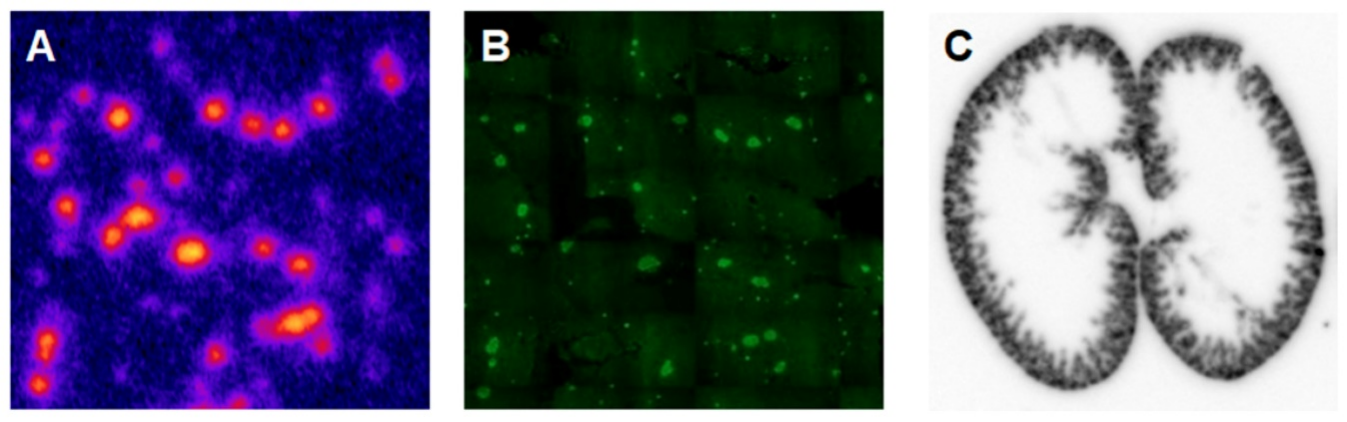
Ex vivo autoradiography of [^177^Lu]Lu-DO3A-VS-Cys^40^-exendin-4 in rat pancreas and kidney. Autoradiograms of the pancreas revealed a heterogenous focal uptake pattern (A), which corresponded to insulin positive islets of Langerhans (B). The renal uptake and retention were localized primarily to the kidney cortex (C).

Another analogue comprising DOTA chelator moiety conjugated to exendin-4 at Cys^40^ position was labeled with ^68^Ga by both manual and automated procedure 46, 58, 70, 93-95. Exendin-4 containing methionine was susceptible to radiolytic oxidation that was suppressed while maintaining relatively high radiochemical yield by fine optimization of the combination of radical scavengers and heating temperature 93. During labeling with ^177^Lu, where the radiolysis is stronger, the stability of [^177^Lu]Lu-DO3A-VS-Cys^40^-exendin-4 was achieved by using smaller amount of ^177^Lu and adding ascorbic acid also to the final product that was stable for at least a week at -20 °C. GLP-1R-mediated uptake of [^68^Ga]Ga-DO3A-VS-Cys^40^-exendin-4 in rat pancreas in vivo was demonstrated by co-administration of cold peptide in excess 58. Moreover, the pancreatic uptake decreased in streptozotocin (STZ) diabetic animals with selectively ablated beta cells. It was possible to clearly distinguish between pancreatic endocrine tumor (INS-1) and pancreatic exocrine tumor (PANC1) xenografts 70. The proximity to the kidneys and diffused shape of the pancreas and difficulty of its anatomical identification precluded in vivo quantification of the uptake reduction in rats. Instead, streptozotocin-induced diabetic pigs were considered for the in vivo studies of GLP-1R as an imaging biomarker of beta cell mass 94. However, no meaningful difference could be detected in the uptake between non-diabetic animals and pigs with STZ induced diabetes with verified complete loss of beta cells 26, 94. Thus, the pancreatic distribution of GLP-1R seems to differ radically in pigs compared to rats, with more expression in the exocrine pancreas. Interestingly, the pigs experienced dose dependently increased heart rate after administration of [^68^Ga]Ga-DO3A-VS-Cys^40^-exendin-4 96, 97 to a degree which usually not seen in other species. Strong pancreatic binding of [^68^Ga]Ga-DO3A-VS-Cys^40^-exendin-4 was also observed in cynomolgus monkeys (NHP). The binding in NHP pancreas was GLP1R mediated, as it could be progressively competed away dose dependently by co-injection of unlabeled DO3A-VS-Cys^40^-exendin-4 58. Using ^177^Lu as a proxy for ^68^Ga in in vitro studies utilizing its higher spatial resolution, it was shown that DO3A-VS-Cys^40^-exendin-4 binds specifically to intramuscularly transplanted islets in mice 9. Thus, DO3A-VS-Cys^40^-exendin-4 radiolabeled with a suitable radiometal is potentially a marker for visualization also of transplanted islets at different sites. Ex vivo autoradiography of sections of explanted pancreata was performed in mouse, rat, pig and NHP after administration of ^68^Ga or ^177^Lu labeled DO3A-VS-Cys^40^-exendin-4. The correlation of binding in the islets was demonstrated by insulin staining of consecutive sections (Figure 1). Interestingly, the islet-to-exocrine ratio varied between species, with the highest contrast - and thus the best promise for pancreatic islet visualization - was seen in rat, followed by mouse and NHP 46. The islet-to-exocrine contrast in pig pancreas was poor, approaching 1 (i.e. similar GLP1R density in the endocrine and exocrine pancreas).

An exendin-4 analogue, wherein the methionine was substituted with norleucine and NODAGA chelator moiety was introduced instead of DOTA (Nle^14^, Lys^40^(Ahx-NODAGA-^68^Ga)NH_2_]-exendin-4), was investigated with the objective to visualize rat pancreatic islets 78. The IC50 value determined in cell was in nanomolar range (2.17 ± 0.42 nM) however the author concluded that in vivo imaging of beta cell in rats could not be achieved, due to strong spill-in of signal from kidneys. The same analogue demonstrated relatively high blood and healthy organ uptake with highest values for GLP-1R positive lung and kidney 1 h post injection in rats 86. Three analogues of exendin-4 comprising NODAGA chelator moiety conjugated to Lys residue at position 12 (^68^Ga-Ex4NOD12), 27 (^68^Ga-Ex4NOD27) or 40 (^68^Ga-Ex4NOD40) where preclinically evaluated with the aim to elucidate the importance of the Lys residues for the biological activity of exendin-4 77. All three analogues showed specific nanomolar binding in CHL-GLP-1R positive cells and respective xenografts in mice, however ^68^Ga-Ex4NOD12 and ^68^Ga-Ex4NOD40 were found preferable.

Exendin-4 analogue, comprising leucine at position 14 and NOTA-conjugated Met-Val-Lys sequence added to Cys^40^ (NOTA-MVK-Cys^40^-Leu^14^-exendin-4), was developed with the objective to reduce kidney uptake 98. It was labeled with ^68^Ga yielding an agent of high affinity determined in INS-1 cell culture and high tumor uptake determined in INS-1 mouse xenografts, with the performance comparable to that of a control agent without cleavable Met-Val-Lys sequence. While the kidney uptake was reduced considerably.

An analogue of exendin-4 functionalized with DFO instead of DOTA, unexpectedly could not be labeled with ^68^Ga at room temperature or at elevated temperature using conventional heating block, but required microwave reactor at 95 °C for 1 min 85. The resulting agent, [Lys^40^(Ahx-DFO-^68^Ga)NH_2_]exendin-4, demonstrated nanomolar receptor affinity, high serum stability and specific in vivo accumulation in nude mice bearing RIN-m5F xenografts. Labeling of [Lys^40^(Ahx-DFO)NH_2_]exendin-4 with ^89^Zr, in contrast to the microwave-assisted labeling with ^68^Ga, could be achieved at room temperature within 2 h, however the quantitative complexation required up to 14-16 h 85. The biological performance of the two analogues was comparable. The longer half-life of ^89^Zr allowed for wider time window for monitoring the biodistribution and revealed long kidney retention time with only 30-40% of the administered radioactivity cleared after 48 h.

Exendin-4 labeled with ^64^Cu using DOTA chelator derivative, ^64^Cu-DO3A-VS-Cys^40^-exendin-4, showed specific uptake in mouse INS-1 xenografts, as well as high uptake in pancreas and liver 79. Two analogues with NODAGA chelator moiety wherein in one analogue the chelator was directly coupled to the peptide and in the other one it was coupled via renal enzyme-cleavable Nε-maleoyl-L-lysyl-glycine (MAL) linker with the aim to reduce the kidney uptake were studied in rats 99. However, the biodistribution in healthy animals were similar in the major organs including kidneys indicating no influence of the MAL linker. Nevertheless, the specific GLP1R-mediated binding to the pancreatic tissue sections was maintained. The DOTA and NOTA based complexes with Cu(II) are not sufficiently stable and more stable cross-bridged chelators have been introduced 100. Bicyclic cage-like chelator (sarcophagine, Sar) forming in vivo stable complex with Cu(II) was used to design monomeric (^64^Cu-BaMalSar-exendin-4) and dimeric exendin-4 (^64^Cu-Mal2Sar-(exendin-4)2) agents 71. The binding affinity determined in INS-1 cells and subcutaneous INS-1 tumor uptake in mice were higher for the dimeric counterpart. Both agents showed high liver and kidney uptake. Bimodal imaging probe based on exendin-4 and bearing Sar chelator moiety for ^64^Cu-labeling and near-infrared fluorescent dye moiety was tested in vivo and ex vivo 101. Specific binding was demonstrated in mice bearing INS-1 xenografts and the pancreatic beta-cell visualization was achieved by both phosphor autoradiography and fluorescent imaging. [Nle^14^, Lys^40^(Ahx-NODAGA-^64^Cu)NH_2_]-exendin-4 comprising norleucine instead of methionine and NODAGA chelator moiety demonstrated GLP-1R mediated binding to islets in rat pancreatic tissue in vitro, however in vivo imaging could not be achieved 78.

### Halogen radionuclide-based analogues

The half-life of 110 min and decay mode with 97% positron emission make ^18^F a very attractive radionuclide. However, most of the ^18^F-labeled exendin analogues demonstrated relatively high non-specific uptake in liver and intestines in animal studies mostly dependent on the labeling methodologies 72-74, 83, 84. Another challenge is the requirement for high specific radioactivity of 200 GBq/µmol 102. The radiolabeling can be accomplished via: conjugation of the peptide to a ^18^F-bearing prosthetic group; click chemistry wherein non-saturated component or tetrazine can comprise ^18^F or be conjugated to the peptide; and peptide comprising a chelator moiety for the complexation with [Al^18^F]^+2^. In the first two procedures an exendin analogue is conjugation to the radiolabeled group and in the latter case the conjugation to the chelator moiety is conducted prior to the labeling with [Al^18^F]^+2^. A potential advantage of using ^18^F as a radiolabel is the faster washout from the kidney cortex, thereby decreasing the local radiation dose as well as enabling less spillover of the signal into the pancreas in rodent models.

**Figure 2:**
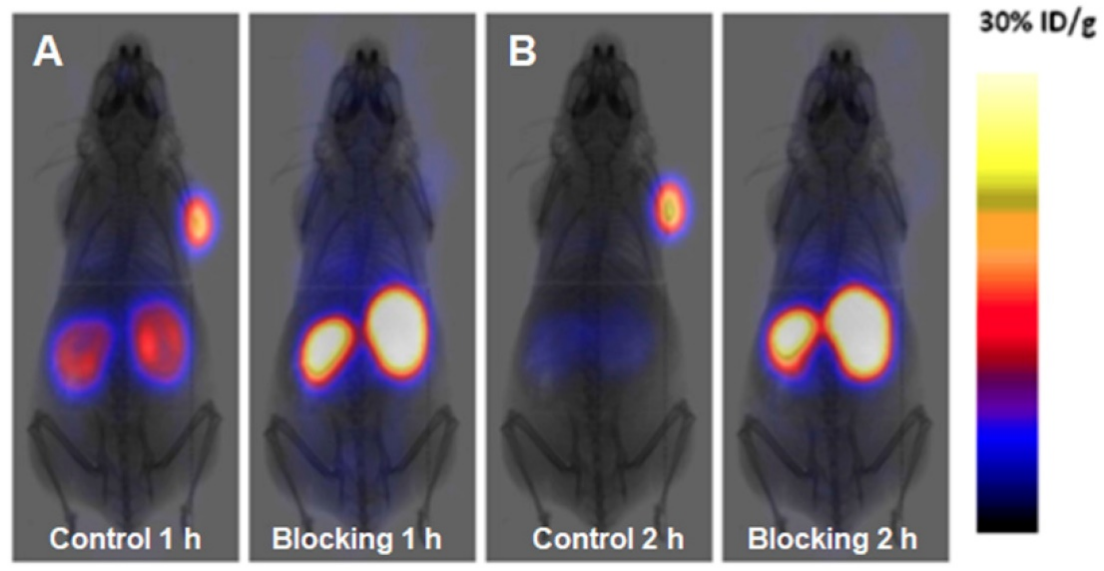
PET images of INS-1 tumor mice at 1 (A) and 2 h (B) post injection of [^18^F]FNEM-[Cys^40^]-exendin-4 (30 μCi) for the control and blocking groups (n = 5/group). Reproduced from 82.

A novel GLP-1 analog, EM3106B, with two cyclic lactam bridges was developed to enhance the biological half-life of the ligand 73, 74. The constrained structure resulted in improved receptor activation capability and resistance against enzymatic degradation. It was labeled with ^18^F via maleimide-based prosthetic group, *N*-2-(4-^18^F-fluorobenzamido) ethylmaleimide (^18^FFBEM) and was used for PET imaging to visualize insulinoma tumors in an animal model. The tracer was tested in nude mice bearing subcutaneous INS-1 insulinoma tumors with GLP-1R and MDA-MB-435 tumors of melanoma origin with low GLP-1R expression. The uptake was correlated with the receptor expression degree. The tracer was excreted both hepatically and renally. Exendin-4 analogue modified with cysteine for site specific labeling via [^18^F]FBEM, [^18^F]FBEM-[Cys^40^]-exendin-4, was studied also in INS-1 xenografted mice 74. The uptake in the tumor was high however abdominal persistent background could complicate the localization of pancreatic uptake. The attachment of [^18^F]FBEM via Cys^39^ of exendin-4 reduced the abdominal background and provided better contrast in mouse xenografts 103. Similar constructs ([^18^F]FPenM-[Cys^40^]-exendin-4 81 and [^18^F]FNEM-[Cys^40^]-exendin-4 82, wherein instead of [^18^F]FBEM prosthetic groups N-5-[^18^F]fluoropentylmaleimide ([^18^F]FPenM) and N-(2-(2,5-dioxo-2,5-dihydro-1H-pyrrol-1-yl)-ethyl)-6-fluoronicotinamide ([^18^F]FNEM) were used, demonstrated comparable biological properties with high tumor uptake and fast liver and kidney clearance 81, 82. Amongst the analogues [^18^F]FNEM-[Cys^40^]-exendin-4 presented advantage of more efficient labeling, and fast kidney clearance wherein the uptake decreased to 2.5 %ID/g within 2 h (Figure 2). Silicon containing exendin-4 was labeled with ^18^F via one-step nucleophilic substitution 104. Visualization of mouse pancreas and xenografts was achieved within 2 h post injection. Four exendin (9-39) analogues were labeled with ^18^F via amino acid residues at positions 9, 12, 27, and 40 and N-succinimidyl-4-[^18^F]fluorobenzoate 105. The analogue labeled at Lys^40^ ([^18^F]FB40-Ex(9-39)) demonstrated the highest uptake in mouse pancreas.

Chelator-mediated Al^18^F-labeling of exendin-4 resulted in an agent, [^18^F]AlF-NOTA-MAL-Cys^40^-exendin-4, with high specificity towards GLP-1R demonstrated in mouse INS-1 xenografts 80. The ex vivo analysis of the plasma and tumor detected intact agent 1 h post injection while content in the kidney and urine was presented by one polar radioactive component. The highest uptake in the tumor was achieved within 5 min while uptake in the kidney continued to increase during 1 h and showed high values that could be reduced by 25% by co-administration of polyglutamic acid solution. However, it was still higher than that of [^18^F]FBEM-[Cys^40^]-exendin-4 and [^18^F]FPenM-[Cys^40^]-exendin-4). [^18^F]AlF-NOTA-MAL-Cys^39^-exendin-4 also demonstrated specific binding in vivo in mouse xenografts and kidney uptake 106 higher than that of ^18^F‑FBEM-Cys^39^‑exendin-4 103.

The insignificance of lysine residue for the binding of exendin-4 to GLP-1R 52 opened possibility for the functionalization of exendin-4 at Lys^12^ position with cysteine conjugated tetrazine for the subsequent labeling with ^18^F-trans-cyclooctene via click-chemistry 107. The resulting imaging agent demonstrated uptake in beta cells in vivo, in mouse models of insulinomas as well as in intestine and kidneys. Another exendin-4 analogue comprising norleucine at position 14 and functionalized with azide at Lys^40^ was labeled with ^18^F via copper-catalyzed click chemistry 108. High and specific uptake was observed in rat pancreatic islets. Construct comprising exendin-4 and ^18^F-fluorobenzoate demonstrated specific binding in vitro in the insulinoma cell line and in vivo in mice bearing insulinoma xenografts 83. The tumor was visualized however the background uptake particularly in the abdominal organs was too high. Lower liver background uptake of ^18^F-tetrazine trans-cyclooctene (TTCO)-Cys^40^-exendin-4 allowed visualization of islet grafts in the liver of islet-transplanted mice 84. The binding specificity was confirmed in INS-1 tumor bearing mice. The kidney uptake was reduced compared to ^64^Cu-labeled analogues.

GLP-1R antagonist, exendin(9-39) was labeled with ^125^I using Bolton-Hunter reagent (^125^I-BH-exendin(9-39)) conjugated to the peptide via lysine residues 109-111. Lysine residues were found critical for the binding of ^125^I-BH-exendin(9-39) to GLP-1R studied preclinically. BH labeling at Lys^19^ position resulted in similar affinities to both mouse and human GLP-1 receptors, while agent labeled at Lys^4^ position detected only mouse GLP-1 receptors. Another analogue of exendin(9-39) with norleucine at position 14 was labeled with ^125^I via Tyr^40^ residue ([Nle^14^,^125^I-Tyr^40^-NH_2_]Ex (9-39)) 112. Despite high affinity, the uptake of [Nle^14^,^125^I-Tyr^40^-NH_2_]Ex (9-39) in INS-1E xenografts in mice was low and transient. In the contrary, the counterpart analogues based on exendin-4 demonstrated high specific uptake in the xenografts. Liraglutide comprising tyrosine amino acid residue was labeled with ^125^I using iodogen method 113. The authors hypothesized that the higher homology with GLP-1 (97%) would provide higher sensitivity and specificity to GLP-1R as compared to exendin-4. The specific binding in lung, tumor and pancreas was observed, however the uptake in the background abdominal organs was also high in nude mice with INS-1 xenografts.

### Structure-activity relationship

Multiple GLP-1 analogues were developed for the structure-activity relationship and enzymatic degradation stability studies. The vast data was thoroughly reviewed previously 15. The importance for specific GLP-1R binding of amino acid residues affected by DPP-IV and NEP was investigated. The sensitive residues were substituted with* D*-amino acids, β-amino acids, and alkylated, glycosylated or halogenated amino acids. Residues with key physicochemical properties were also substituted or functionalized for improved in vivo stability and renal clearance as well as for radioactive labeling. The influence of the chelator position in exendin-4 on the binding and biodistribution of the agents was studied in vitro in GLP-1R transfected cells and ex vivo in mice bearing CHL-GLP-1R positive tumor 77. The analogues with chelator (NODAGA) conjugated via lysine at position 12, 27 or C-terminus maintained their binding specificity with comparable affinity. The modification at position 27 was considered less preferable. The binding specificity was deteriorated when exendin(9-39) was labeled with ^18^F via Lys^27^ 72. The computational investigation of exendin-4 interaction with GLP-1R in the presence of water using MembStruk method demonstrated the importance of both lysine residues for the binding affinity of the ligand 114. However, the modification at Lys^12^ position, [Lys^12^(^111^In-BnDTPA-Ahx)]exendin-4, did not deteriorate the binding capability 89.

### Radiolytic stability

Exendin-4 contains methionine and tryptophan amino acid residues that are prone to oxidation especially under labeling conditions using high amount of radioactivity and elevated temperature. This results in formation of oxidized by-product compromising the purity of the radiopharmaceutical. The change in biological activity, in particular receptor binding affinity upon the oxidation can be expected and it is an important task to investigate the issue. Exendin-4 based analogues, comprising methionine or norleucine or oxidized methionine amino acid residue and labeled with ^99m^Tc were generated and their physicochemical and biological properties were investigated 86. Oxidized product maintained binding capability though to somewhat lesser extent as compared to the intact counterpart. Although the oxidized form was more hydrophilic, the binding capacity was comparable to that of non-oxidized counterpart.

The radiolytic oxidation can be suppressed by radical scavengers such as ethanol, ascorbic acid, gentisic acid, HEPES, selenomethionine, sodium thiosulfate, L-methionine, etc. 77, 93, 115. The concentration of the radical scavengers requires optimization, e.g. higher amount of ascorbic acid deteriorates the radiolabeling with ^68^Ga, while gentisic acid shows less influence on the reaction efficiency 93. Another factor that impacts the extent of the radiolysis is the peptide precursor concentration 93. Increased concentration may decrease the radiolysis. However, complete elimination of the oxidized product is difficult to achieve since the precursor solution might contain the oxidized form, building up during the storage, prior to the labeling. The stability of dry HYNIC-Met^14^-Exendin-4 during the storage was improved in the presence of L-methionine 86. In the best-case scenario, a single radiochemical entity is preferred. However, the comparable binding capacity would allow the calculation of the radiochemical purity as a sum of the two components. Nevertheless, attempts to decrease the formation of the oxidized product must be conducted until the options are exhausted.

Another solution to improve the radiolytic stability of exendin analogues was the substitution of methionine with its isosteric analogue, norleucine 78, 86, 116. The replacement of methionine by norleucine improved the binding capability 86. The biodistribution pattern of the analogues was similar. Exendin-4 contains also Trp however the corresponding by-products have not been investigated yet and given the fact that after the substitution of the Met with Nle the labeling results in a single product it is plausible that the oxidation of Trp under those conditions does not occur.

### Specific radioactivity

Specific radioactivity (SRA) can in general terms be defined as concentration of a radioactive material in a sample (e.g. Bq/mol). The importance of SRA of an imaging agent for enabling high contrast imaging of high affinity/specificity targets and adequate quantification of the target expression as well as to reduce pharmacological and toxic effects is an established knowledge 117. It is particularly crucial in the case of in vivo beta cell imaging and quantification wherein the density of GLP-1R is rather low and the agonist ligands are of high potency. Preclinical studies, investigating imaging agent uptake as a function of administered total peptide dose, demonstrated that the total amount of exendin-3/exendin-4 peptide that can be administered to mice and rats in order to provide adequate imaging of pancreatic beta cells should not exceed 20 pmoles 58, 63, 68. Optimal targeting of subcutaneous INS-1 tumors in BALB/c nude mice corresponded to less than 0.1 μg of [Lys^40^(^111^In-DTPA)]Exendin-3 68. The highest pancreatic uptake in rats corresponded to 0.1 μg/kg of ^68^Ga-DO3A-exendin-4 58. It is possible that even lower mass doses may have yielded higher pancreatic uptake, but the specific radioactivity (SRA) in combination with the small size of the rats limited the minimal doses to 0.1 μg/kg. Finally, dose escalation studies in NHP demonstrated optimal pancreatic binding of ^68^Ga-DO3A-exendin-4 for injected peptide mass doses below 0.2 µg/kg. The peptide mass should be associated with radioactivity amount that would provide statistically sufficient counts for detection putting demand on SRA value.

In order to enhance SRA and enable detection and quantification of small changes in beta cell mass responsible for diabetic pathophysiology progression, a number of exendin-3 analogues carrying one, two, or six DTPA moieties were developed 118. The analogues were labeled with ^111^In and the one comprising six chelator moieties demonstrated 7-fold increase in SRA. It maintained its biological activity towards GLP-1R and demonstrated enhanced radioactivity counts in mice and rats with 3-fold improvement of the image contrast and pancreas visualization.

### Animal models

The biological evaluation of various agents was performed using cell cultures (INS-1, islets), tissue section autoradiography (pancreas, INS-1 xenograft sections), ex vivo and in vivo biodistribution in healthy animals (mouse, rat, pig, non-human primate) and animal models of metabolic disease including nonobese diabetic (NOD) mice, ob/ob mice, biobreeding diabetes-prone rats, Zucker diabetic fatty rats, alloxan, diphtheria toxin or STZ induced diabetes in rodents and pigs.

The possibility of in vivo longitudinal imaging of implanted islets is of utmost importance not only for monitoring the survival and function maintenance of engrafted islets but also for the adjustment of immunosuppressive regime and assessment of novel transplantation sites. Revascularization is essential for the survival of the engrafted islets following transplantation, and for the tissue perfusion and accessibility of the intravenously administered imaging agents and therapeutics. Targeting GLP-1R was demonstrated relevant for the in vivo imaging. The correlation was found between the formation of the microvasculature in transplanted islets and the uptake of [Lys^40^(DTPA-^111^In)]-exendin-3 in mice the islets transplanted into the calf muscle 119. The intra-islet vasculature was perceptible after 2 weeks and grew further within 6 weeks of the study penetrating from the periphery into the core of the transplant. Preclinical ex vivo study demonstrated feasibility of beta-cell mass quantification in intramuscular islet grafts in mice using [^177^Lu]DO3A-VS-Cys^40^-exendin-4 9. Linear correlation between the radioactivity uptake and the number of transplanted islets was found. The islet-to-background signal ration was high (40) and the binding in individual islets was similar to that of pancreatic islets.

Human islets intraportally transplanted into NOD/SCID mouse livers via portal vein (i.e. currently the clinically relevant site) were visualized by ^64^Cu-DO3A-VS-Cys^40^-exendin-4 79 and ^18^F-TTCO-Cys^40^-exendin-4 84 and the uptake correlated with the number of the transplanted islets. The uptake of ^177^Lu-labeled analogue, [^177^Lu]DO3A-VS-Cys^40^-exendin-4, was correlated with gradually increasing number of islets ingrafted into the abdominal muscle of nondiabetic mice thus demonstrating potential for the in vivo quantification of beta cell mass 9. The high resolution of the ex vivo tissue autoradiography images allowed accurate correlation of the ^177^Lu signal with insulin location determined by immunohistochemistry.

^111^In-labeled exendin-3 was used to determine beta cell mass in mouse and rat models for spontaneous T1D, and demonstrated reduced uptake as compared to the healthy animals 120. Interestingly, the uptake in the exocrine pancreas was relatively higher in mice compared to rats. Exendin-3 analogue labeled with ^111^In, [Lys^40^(^111^In-DTPA)]exendin-3, was tested in a rat model of alloxan-induced beta cell loss 57 and diphtheria toxin induced beta loss in RIP-DTR mouse model 121 wherein the in vivo uptake correlated with beta-cell mass. A rat model of alloxan-induced beta cell loss was found to have less exocrine background binding as compared to the mouse model 122. However, it is not clear which model that best translate to the human situation and the binding of exendin-4 in the human exocrine pancreas. The accumulation of [Lys^40^(^111^In-DTPA)]exendin-3 in the exocrine pancreas in mice was argued to be mediated by receptors other than GLP-1R, but this was based om mRNA transcription analysis of isolated islet and exocrine pancreatic compartments, rather than GLP1R density assessment. Conversely, in a recent study by Khera and colleagues, it was demonstrated that exendin-4 binding is indeed mediated exclusively by GLP1R expression in the exocrine pancreas in mouse 123.

**Figure 3:**
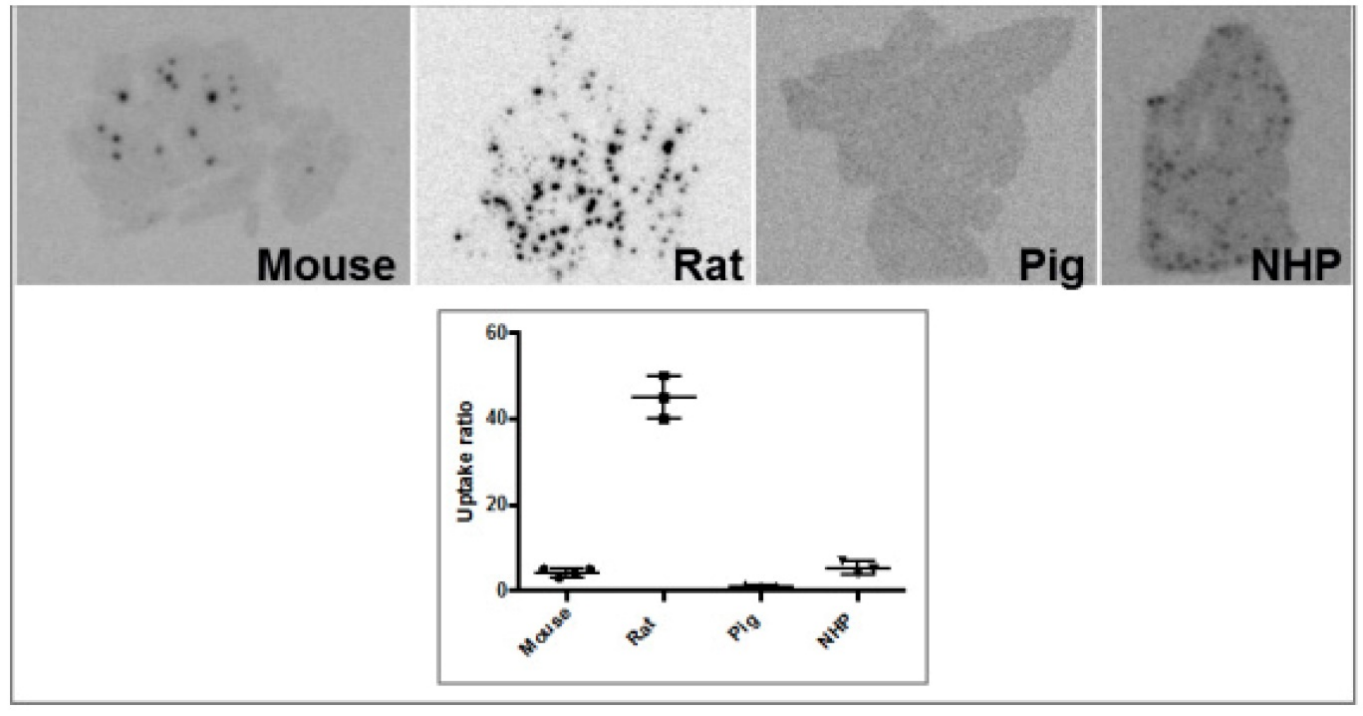
Ex vivo autoradiograms of pancreas in vivo distribution of [^177^Lu]Lu-DO3A-VS-Cys^40^-exendin-4 in mouse, rat, pig, and non-human primate. The islet contrast (graph) defined as the islets-to-exocrine pancreas (IPR, Uptake ratio) ratio was highly dependent on the species (mouse=4.3±1.0, rat=45±5, pig=1.1±0.2, NHP=5.3±1.5), mainly reflecting the difference in background binding. Error bars represent standard deviation (n=3-4).

Brown Norway rat model was found optimal due to the favorable pancreas-to-background uptake ratio. The uptake of [^64^Cu]-DOTA)NH_2_-exendin-4 in islet cells was considerably reduced in Zucker diabetic fatty rats 124. On the other hand, the uptake of [^68^Ga]DO3A-VS-Cys^40^-exendin-4 was increased in pancreatic islets in mice with mutations in the MEN1 tumor suppressor gene 125. Radiolabeled exendin-4 therefore seems to be able to distinguish between a large continuous spectrum of aberrantly regulated beta cells, from low binding in islets in T1D/ T2D, via normal uptake to unaffected beta cells, to somewhat increased binding in MEN1 deficient islets onto strong binding in insulinoma cells. This is particularly evident in mouse and rat islets, and it is crucial to take into consideration the biological differences in pancreatic distribution of GLP1R amongst various species, when interpreting the aforementioned results (Figure 3). Importantly, it is currently not clear which animal model best capitulates the situation in the human exocrine pancreas. Different GLP-1R directed polyclonal antibodies have produced variable results regarding the existence or extent of GLP1R in exocrine pancreas or pancreatic ductal cells 43, 45. In most studies, beta cells exhibit strong antibody staining. Single cell or small cell clusters close to the ductal epithelium were identified as strongly GLP1R positive and these cells were frequently also insulin positive. Kirk et al also found a relevant proportion of exocrine cells to be GLP1R positive, but with an intensity a third of the beta cells 45. This islet-to-exocrine ratio of 3 is incidentally in line with the ratios seen in mouse (islet-to-exocrine ratio 4.3) and NHP (islet-to-exocrine ratio 5.3) as determined by *ex vivo* autoradiography with [^177^Lu]Lu-DO3A-VS-Cys^40^-exendin-4 (Figure 3) 46. These studies taken together indicate that mouse and NHP animal models may constitute suitable approximations of the expression of GLP1R in human exocrine tissue. Additionally, this indicates that the residual pancreatic signal seen for [Lys^40^(Ahx-DTPA-^111^In)NH_2_]-exendin-4 also in human subjects with long-standing T1D is due to the exocrine binding to GLP1R 57. Assuming that the exocrine signal constitutes an obstacle for accurate visualization of the beta cells, Khera et al suggested to pretreat mice with lipophilic Cy7-exendin4 to preblock especially the exocrine population of GLP1R. After the pretreatment, sufficient amount of GLP1R remained on the beta cells, which then could be imaged with fluorescent or ^111^In-exendin-4 123.

Moreover, GLP-1R was considered as a biomarker to assess its cardioprotective effect of attenuation of myocardial inflammatory response and fibrosis after ischemic injury 126, 127. Myocardial ischemia and reperfusion (MI/R) rat models were used. The rapid enhancement of GLP-1R expression upon ischemia-reperfusion was detected using ^18^F-FBEM-Cys^40^-exendin-4 in rats 126. The results open possibility for the optimization of the therapeutic intervention time schedule. Kinetic modelling of the enhanced uptake of ^68^Ga-NODAGA-exendin-4 in the infarcted area in disease model rats revealed irreversible binding and correlated with the presence of macrophages involved in the MI healing process 127.

### Physiological potency and GLP1R antagonists

Most commonly used GLP-1R targeting radioimaging agents are based on exendin-3 and exendin-4 peptides that present some issues such as high potency of the agonist inducing hypoglycemia 26, 66, 75. In the case of potent ligands such as exendin-4 the amount that can be administered without induction of pharmacological effect can be very limited, however, if high amount of the peptide must be injected for various technical reasons, the episodes of severe hypoglycemia can be prevented by continuous infusion of glucose 38. Another strategy to solve the problem of side effects is to use radiolabeled antagonists, and preclinical studies using agents based on exendin(9-39)-amide isolated from Heloderma suspectum venom have been conducted 67, 109, 128. The GLP-1R targeting properties of ^125^I-Bolton-Hunter conjugated Ex(9-39)NH2 (^125^I-BH-exendin(9-39)) were confirmed both in vitro and in vivo in mouse 109. Further investigation demonstrated that the number of binding sites was not higher for the antagonist ^125^I-BH-exendin(9-39) as compared to the agonist 111. The authors also demonstrated the influence of the BH labeling site on the targeting properties, in particular, BH labeling on Lys^19^ resulted in the agent with similar affinities to both rat and human GLP-1 receptors, while agent labeled at Lys^4^ detected only rat GLP-1 receptors. Pharmacokinetics of ^125^I-BH-Ex(9-39)NH_2_ studied in nude mice bearing rat Ins-1E tumors demonstrated low kidney uptake and fast blood clearance, however the uptake in tumor also decreased by 50% within 4 h 128. Another antagonist analogue, [Lys^40^(DTPA-^111^In)]exendin(9-39), was compared to the agonist agents [Lys^40^(DTPA-^111^In)]exendin-3 and [Lys^40^(DTPA-^111^In)]exendin-4 67. All three agents exhibited similar IC50 values in cell culture, however antagonist [Lys^40^(DTPA-^111^In)]exendin(9-39) demonstrated low specific uptake with fast washout in vivo in mouse xenografts. The introduction of chelator moiety at Lys^27^ instead of Lys^40^ did not improve the binding characteristics of antagonist [Lys^27^(Ahx-DOTA-^68^Ga)]Ex(9-39)NH_2_ and [Lys^27^(NODAGA-^68^Ga)]Ex(9-39)NH_2_ 128. The authors found these candidates not suitable for imaging of the GLP-1 receptor expression in vivo. Labeling with ^125^I of antagonist exendin(9-39) at terminal Tyr^40^, [Nle^14^,^125^I-Tyr^40^-NH_2_]Ex (9-39), also did not improve the tumor accumulation in mice despite recognition of larger number of binding sites 112. The uptake in pancreatic beta cells and insulinomas was found species dependent for another antagonist, ^125^I-BH-exendin(9-39), in particular no binding was observed in human tissue 110. Antagonist, [^18^F]FB40-Ex(9-39), visualized mouse pancreas within 30 min post injection with moderate pancreas-to-organ ratio 105.

### Kidney uptake reduction

The major difficulties of the accurate localization and quantification of the beta cells in vivo in rodents, in particular is the proximity to the left kidney and the irregular shape of the pancreas that cannot readily be accurately identified by CT. In the preclinical setup using mice and rats, nephrectomy provides the solution, even if this precludes longitudinal imaging also in animals, e.g. baseline and follow-up scans in treatment studies. The exploration of other options lead to the development of dual tracer methodology wherein additional agent with high accumulation in the exocrine pancreas and low kidney uptake is used for the accurate delineation of the pancreas 121. In particular, the combination of [Lys^40^([^111^In]DTPA)]exendin-3 and 2-[^123^I]Iodo-L-phenylalanine used in RIP-DTR mice demonstrated more accurate quantification of beta cells that correlated with ex vivo autoradiography results. In order to exclude necessity for the nephrectomy and additional probes, an image analysis method was developed and characterized 129. The measurement of ROIs with 40% cutoff allowed reliable estimate of pancreatic uptake in vivo by SPECT/CT and ^111^In-labeled exendin-4 in mice 129.

The clinical relevance of the GLP-1R targeting radioactive agents might be hindered by the potential high radiation dose to the radiosensitive kidneys and the understanding of the uptake mechanism would allow development of means for the uptake reduction. Generally, peptides present renal excretion, and a common drawback of the metal radionuclide labeled exendin analogues used in the clinical studies is high kidney uptake. The high kidney uptake additionally presents a problem with respect to the imaging accuracy of adjacent pancreatic tail, especially in SPECT due to the intrinsically lower resolution in clinical scanners as compared to PET. It is essential to decrease the uptake not only for the accurate detection and quantification of the target of interest but also from the dosimetry and radiotherapeutic point of view. The administered therapeutic radioactivity dose is very often limited by the renal retention and consequently high absorbed dose to the kidneys potentially could compromise kidney function. The high radiation dose may lead to renal failure and uremia. Mice receiving high kidney absorbed dose from ^111^In-DTPA-exendin-4 (>40 Gy) developed long-term kidney damage in tubular and glomerular compartments 130.

Although GLP-1R mRNA was identified in the kidneys previously 131, the uptake of ^111^In-DTPA-Lys^40^-exendin4 could not be precluded by excess of non-labeled ligand 62 and was higher compared to the radioiodinated analogues. The target accumulation of radioactivity using ^111^In-DTPA-Lys^40^-exendin-4 was found superior to radioiodinated peptides in terms of sensitivity and specificity 62. Ex vivo autoradiography of rat kidney frozen sections using [^177^Lu]-DO3A-VS-Cys^40^-exendin-4 59 revealed high uptake localized in the cortex indicating that most likely the radioactivity retention occurred due to tubular reabsorption of the peptide 132. The renal function in rats was not compromised by acute administration of 50 MBq/kg [^177^Lu]-DO3A-VS-Cys^40^-exendin-4 according to the blood creatinine level. Despite the notion of GLP-1R expression in renal cortex, the uptake of [^68^Ga]-DO3A-VS-Cys^40^-exendin-4 could not be precluded by the excess of co-administered exendin-4 26, 58. More studies demonstrated that the vast majority of renal uptake was not GLP-1R mediated since it was not possible to block the uptake by the excess of unlabeled analogues 68, 86. It is still possible that the kidneys present some GLP1R mediated binding of radiolabeled exendin-4, but this would be negligible in comparison to the uptake due to reabsorption according to the available literature.

Various agents partially precluding renal peptide reabsorption, e.g. arginine, lysine, gelofusine, and sodium maleate were suggested. The effect of *D*- and *L*-lysine on the renal uptake reduction was thoroughly investigated preclinically and clinically for antibodies and antibody fragments 133 indicating that the positively charged amino groups neutralize the negative charge of the luminal tubular cell surface thus precluding reabsorption of protein/peptide molecules. The co-administration of *L*-lysine and/or *L*-arginine became an integrated part of peptide receptor radionuclide therapy in neuroendocrine tumors 134. However, in case of exendin analogues the results were not encouraging most probably due to the negative charge of the exendin-3 peptide moiety 135.

Preclinical studies have been conducted to investigate the kidney uptake mechanism, and megalin mediated reabsorption mechanism in combination with metabolic trapping was hypothesized 62. A natural megalin ligand, albumin and its fragments were investigated precluding the uptake of ^111^In-exendin in rat kidneys by 52% wherein lysine and gelofusine reduced the kidney uptake, respectively by 15 and 25% 136. Furthermore, in vivo studies using megalin-deficient mice demonstrated lower kidney uptake of ^111^In-DTPA-exendin-3 analogue compared to wild-type mice indicating binding to megalin receptor with subsequent internalization and lysosomal entrapment as the mechanism of kidney uptake and retention 135. The extent of the uptake reduction was different for male and female mice with respective values of 62% and 52%, and interestingly it was also dependent on the administered peptide mass with higher reduction values for the higher peptide doses presumably indicating higher specificity of the agent towards megalin receptors. Repeated administered dose of 40-50 MBq of ^111^In-DTPA-exendin-4 resulted in 70 Gy kidney absorbed dose in wild-type mice while in megalin-deficient mice was it 20-40 Gy 130.

It was hypothesized that the renal reabsorption is influenced by the number of charged amino acids and their distribution over the peptide chain 137. Kidney uptake of [Lys^40^(Ahx-DOTA-^68^Ga)NH_2_]-exendin-4 was reduced by pretreatment with positively charged poly-glutamic acid (PGA, 49%) or the plasma expander Gelofucine (succinylated gelatin, 60%). A combination of PGA and Gelofucine decreased the renal uptake even further (78%) 69, 137. The kidney uptake of ^111^In-DTPA-Lys^40^-exendin-4 in rats was also reduced by either PGA (29%) or Gelofusine (19%) used separately, however their synergetic effect was the highest causing 48% uptake reduction 137. Interestingly, anionic amino acid Lysine did not affect the kidney uptake in rat, indicating that exendin-4 is taken up in the kidneys by a mechanism different from that of somatostatin analogues. The promising preclinical results on renal uptake reduction using plasma expanders was recently partly confirmed in a clinical study in healthy volunteers, where gelofusine reduced the renal uptake of ^111^In-DTPA-Lys^40^-exendin-4 by almost 20%, while not impacting the pancreatic binding 138. Thus, interpolating these results to exendin-4 peptide receptor radionuclide therapy (PRRT) using lutetium-177 as label, the authors estimate that the amount of ^177^Lu-exendin-4 could be increased accordingly without reaching the 23Gy limit in kidney. In a simulation, insulinomas could be exposed to up to 156 Gy which is in range for doses inducing tumor shrinkage by DOTATATE PRRT. Furthermore, the intervention improved delineation of the pancreatic tail allowing improved assessment of GLP1R density.

The hypothesis of the involvement of megalin and cubilin receptors in the renal reabsorption was tested by using derivatives of albumin, a natural ligand to megalin and cubilin receptors 136, 139. Fragments of albumin, derived from the digestion of albumin by cyanogen bromide, with various charges were studied. The biodistribution of the fragment of 36 AA and -3 net charge in rats demonstrated inhibition of [Lys^40^(Ahx-DTPA-^111^In)NH_2_]-exendin-3 reabsorption by 26% with no effect on any other organ uptake, and no adverse effects.

The kidney retention is influenced by the radionuclide labeling chemistry and the difference in the physicochemical properties of the radiolabeled catabolites. For example, lysosomal degradation of a protein/peptide radioiodinated directly at tyrosine yields lipophilic catabolites of iodinated tyrosine that leave the tubule cell. While degradation of proteins/peptides either radioiodinated via prosthetic groups or radiometalated via chelator moiety results in hydrophilic and charged radioactive catabolites that get trapped insight the cell. The feasibility of tuning of kidney uptake by using halogen radiolabels has been studied. The kidney uptake decrease could also be achieved using Ex(9-39)NH2 antagonist analogues labeled with non-residualizing ^125^I moeity 128, 140 and ^18^F 74, 81. Exendin-4 analogue labeled with ^125^I via tyrosine residue was reported to drastically decrease the kidney uptake to only 7.5±0.7%IA/g 112 or 3.3±0.6%IA/g 141. The ratio of tumor-to-kidney investigated in mice with insulimona cell xenografts was 50 times higher for [Nle^14^,^125^I-Tyr^40^-NH_2_]exendin-4 (9.7) as compared to [Nle^14^,Lys^40^(Ahx-DOTA-^68^Ga)-NH_2_]exendin-4 (0.2). However, protection of thyroid was required, and was reduced by 94% using irenat. The authors attributed the low kidney retention to the in vivo deiodination of the agents. The improvement of tumor-to-kidney ratio for ^125^I-BH-Ex(9-39)NH_2_ was 20-fold as compared to [Nle^14^, Lys^40^(Ahx-DOTA-^68^Ga)NH_2_)]Ex-4 128. The drawback of using ^125^I-BH-Ex(9-39)NH_2_ was high accumulation in the thyroid, however it could be considerably reduced by inhibitor of the sodium iodide symporter (e.g. irenat) 142, 143. Exendin-4 labeled with ^18^F (^18^F-TTCO-Cys^40^-exendin-4) demonstrated considerably lower kidney uptake as compared to radiometal-labeled counterparts 84. The fast renal clearance was also demonstrated for another ^18^F-labeled analogue, [Nle^14^,Lys^40^]-[^18^F]exendin-4 108. Silicon containing exendin-4 labeled with ^18^F 104 and antagonist, [^18^F]FB40-Ex(9-39) 105 also demonstrated lower kidney uptake and retention comparable to radiometal labeled agents. On the other hand, it should also be noted that the uptake in GLP-1R rich target tissues (e.g. pancreas or insulinoma) of radio-halogenated exendin analogues may similarly be decreased by the lack of a radionuclide trapping mechanism.

Another possible reason for the high kidney uptake is the metabolism of the exendin analogues, and final elimination of the catabolites by kidney 144. Improved stability of exendin-based agents could potentially decrease the kidney uptake and absorbed dose. It was demonstrated in pigs that the degradation of GLP-1 was influenced by NEP, and inhibition of NEP and dipeptidyl peptidase IV (DPPIV) *in vivo* could improve metabolic stability of the ligand 145. However, the improvement could be predicted to be minor, given that one of the primary reasons for the development of exendin-4 as a therapeutic GLP-1R agonist, was its resistance to DPPIV as compared to endogenous GLP-1.

An alternative approach was to introduce a metabolizable linkage Nε-maleoyl-L-lysyl-glycine (MAL) into an exendin-4 analogue ([^64^Cu]NODAGA-MAL-exendin-4) 99. The novel agent maintained the biological activity and demonstrated specific uptake in rat pancreatic islets, however kidney uptake was not reduced compared to [^64^Cu]NODAGA-exendin-4 without the linkage. A cleavable substrate for meprin β protease expressed in the kidney brush-border membrane was introduced between the binding moiety of exendin-4 and ^111^In-NODAGA moiety 146. The biodistribution in nude mice bearing CHL-GLP-1R positive xenografts showed specific accumulation in the tumor cells despite the introduced modification. Recombinant meprin β efficiently digested the linker sequence in in vitro assay. However, the kidney uptake in vivo was comparable to that of the reference agent, ^111^In-NODAGA-exendin-4. The authors hypothesized that the peptide uptake was most probably faster than the cleavage of the linker.

**Figure 4:**
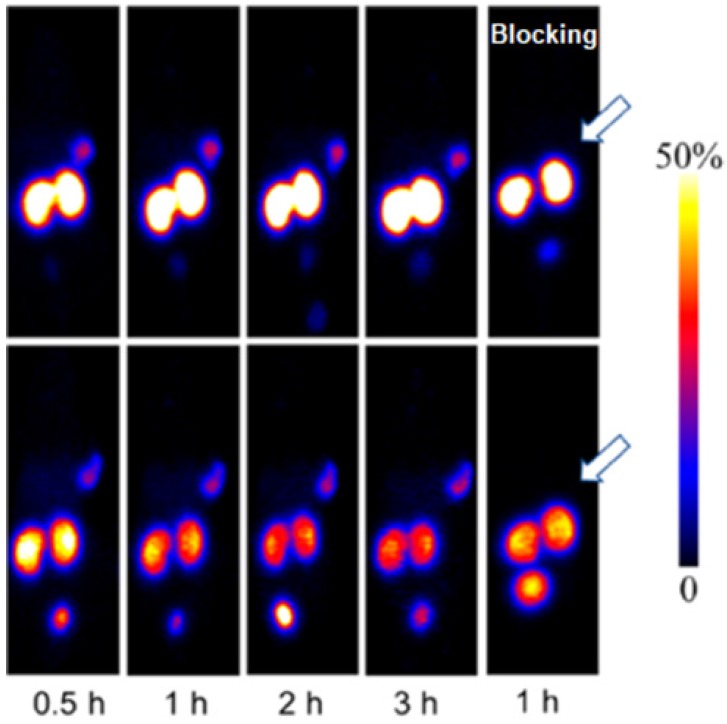
MicroPET images of INS-1 tumor mice at different time points after intravenous injection of ^68^Ga-NOTA-Cys^40^-Leu^14^-exendin-4 (upper panel) and ^68^Ga-NOTA-MVK-Cys^40^-Leu^14^-exendin-4 (lower panel). Reproduced from 98.

^68^Ga-NOTA-MVK-Cys^40^-Leu^14^-exendin-4 comprising cleavable Met-Val-Lys (MVK) sequence demonstrated remarkable reduction in kidney uptake compared to ^68^Ga-NOTA-Cys^40^-Leu^14^-exendin-4 while retaining high accumulation in GLP-1R expressing INS-1 mouse xenografts (Figure 4) 98. Presumably, the agent was cleaved by brush border membrane enzyme on kidneys to ^68^Ga-NOTA-Met-OH that was rapidly excreted.

## Clinical accomplishments

A number of clinical research studies has been performed since the first study on two patients with insulinoma using [Lys^40^(Ahx-DTPA-^111^In)NH_2_]-exendin-4 (DTPA: diethylenetriamine tetraacetic acid) for the imaging of GLP-1R 64 and nowadays several multicenter clinical trials using various GLP-1 analogues are ongoing in Europe 26, 37, 64-66, 147-149. Exendin-4 analogues that are relatively stable agonists of GLP-1R labeled with gamma emitting radionuclides such as ^111^In and ^99m^Tc demonstrated high sensitivity in GLP-1R imaging and insulinoma detection with SPECT 65, 66, 150. PET technique offers further advantages of higher sensitivity and spatial resolution as well as accurate quantification. These advantages are crucial especially considering the small size of insulinomas. Such positron emitting radionuclides as ^18^F, ^64^Cu, ^68^Ga, and ^89^Zr have been used offering both advantages and drawbacks of their physical and chemical characteristics.

### SPECT/CT

Exendin-4 ligand modified with either DTPA or DOTA at lysine amino acid residue and labeled with ^111^In resulting in [Lys^40^-(Ahx-DTPA-^111^In)NH_2_]-exendin-4 and [Lys^40^-(Ahx-DOTA-^111^In)NH_2_]-exendin-4 demonstrated prominent detection of insulinomas that could not be unambiguously localized by conventional radiological methods 64, 66. The localization of the lesion enabled successful guided surgery in both patients 64, and the delineation of benign insulinomas was accomplished in six patients 66 wherein morphological diagnostic methods were conclusive in four out of six cases. Moreover, the long physical half-life of ^111^In allowed the subsequent resection of the tumor mass by radioguided surgery using γ-probe intraoperatively 66. The overexpression of GLP-1R in the resected lesion tissue was confirmed by vitro autoradiography. The potential of [Lys^40^-(Ahx-DTPA-^111^In)NH_2_]-exendin-4 SPECT/CT for the improved patient management was investigated in a prospective study with 11 patients affected by malignant insulinoma 37. The patients were also examined with ^68^Ga-DOTATATE PET/CT for the detection of SSTR expressed in high density in malignant insulinoma. The authors concluded that in contrast to benign insulinomas, malignant insulinomas often lack GLP-1 receptors while express SSTR type 2 more often. A subsequent larger study with 30 patients demonstrated that [Lys^40^-(Ahx-DTPA-^111^In)NH_2_]-exendin-4 SPECT/CT was more sensitive diagnostic technique than conventional CT/MRI in detection of insulinomas and it changed therapeutic management of patients affected by endogenous hyperinsulinaemic hypoglycemia 150. These successful studies also pointed out the limitation of the low spatial resolution of ^111^In/SPECT and interference of the high kidney uptake with detection of lesions in pancreas regions close to the kidney. The adequate localization required a second SPECT examination 3-7 days later after the sufficient clearance of the kidneys from the radioactivity.

Five healthy volunteers and five patients affected by T1D were engaged in a study using ^111^In-DTPA-exendin wherein significant reduction (60%) of the integrated radioactivity uptake in the pancreas (i.e. radioactivity concentration multiplied with pancreas volume) was observed in the patients 57. Despite high interindividual variation, the separation of the two groups was distinguishable. However, the radioactivity concentration of ^111^In-DTPA-exendin in the pancreas was overlapping between the healthy controls and the subjects with T1D, suggesting that the atrophy of the pancreas in long standing T1D accounted for the majority of the decreased integrated uptake 54. Further, these results indicate binding of ^111^In-DTPA-exendin in the pancreas of subjects with T1D in the range of the healthy controls. This surprising finding was suggested to indicate evidence of a population of residual GLP-1R expressing beta cells long after T1D debut. Another source of the signal may occur from binding of ^111^In-DTPA-exendin to other GLP-1R positive cell types in the pancreas, which has been shown to vary considerably between species 46. These and other outstanding questions are addressed by an ongoing clinical trial where ^111^In-DTPA-exendin is administered prior to planned removal of part of the pancreas, where the autoradiographic uptake pattern in pancreatic sections will be correlated to the islet distribution (NCT03889496). The reduction of kidney uptake of ^111^In-DTPA-Lys^40^-exendin-4 by 18.1% was achieved in a clinical study with ten healthy volunteers using Gelofusine 138. The procedure even allowed for better discrimination of the pancreatic tail without reduction of the pancreatic uptake. Importantly, in relation to potential radiotherapy applications, the procedure yielded an improved dosimetric profile. Exendin-4 based imaging in metabolic disease has otherwise shifted towards ^68^Ga based-PET imaging, which is outlined in detail below.

The lower γ-energy and shorter half-life of ^99m^Tc as compared to ^111^In could improve the quality of images and considerably reduce the radiation burden to the patient and medical staff. The ready availability of ^99m^Tc from a generator system provides another crucial advantage. The respective agent, [Lys^40^(Ahx-HYNIC-^99m^Tc/EDDA)NH_2_]exendin-4, was used in a study of 11 patients with negative results on conventional diagnostic imaging methods 65. The sensitivity and specificity of [Lys^40^(Ahx-HYNIC-^99m^Tc/EDDA)NH_2_]exendin-4 SPECT/CT were assessed to be 100% in patients with benign insulinoma. In one patient out of two with malignant insulinoma the lesion was found only in the region of local recurrence. In the subsequent study 39 forty patients with hypoglycemia were examined with [Lys^40^(Ahx-HYNIC-^99m^Tc/EDDA)NH_2_]exendin-4 SPECT/CT and positive results were observed in 28 patients. The high kidney uptake presented similar complications as in the case of ^111^In-labeled analogues and the optimal imaging time in terms of pancreatic lesion localization was determined as 5-6 h post injection. [Lys^40^(Ahx-HYNIC-^99m^Tc/EDDA)NH_2_]exendin-4 SPECT/CT was also successfully used for the diagnostic detection of medullary thyroid cancer 151.

### PET/CT

Clinical PET scanners offer advantages over SPECT in terms of higher spatial resolution and sensitivity, accurate quantification of the tracer uptake and consequently target concentration as well as possibility for dynamic scanning and subsequent kinetic modeling and uptake mechanism investigation. Digital detectors introduced to the new generation of PET/CT and PET/MRI scanners increase the throughput, improve sensitivity and resolution making the PET technique even more attractive. Positron emitting 68Ga is a very attractive radionuclide in terms of its ready availability from a simple generator system as well as cyclotron, straightforward labeling chemistry, and favorable decay characteristics 117. In the context of theranostics, ^68^Ga is particularly interesting, as it forms a diagnostic/therapeutic radionuclide pairing with ^177^Lu, with which it shares critical features such as the ability to form stable complex with DOTA.

**Figure 5:**
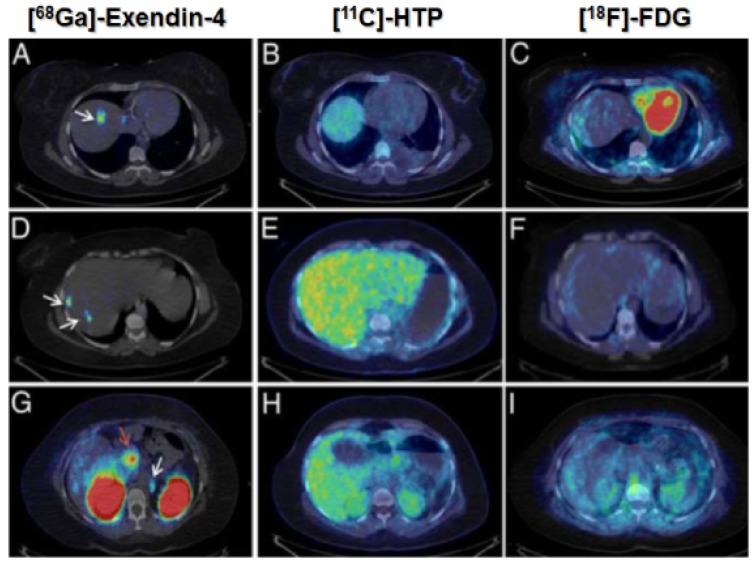
[^68^Ga]Ga-DO3A-Exendin-4/PET-CT revealed several GLP-1R positive lesions (white arrows) in the liver (A, D) and a paraortallymph node (G). Beta cells in normal pancreas (red arrow) have significant expression of GLP-1R and can also be visualized by this technique (G). No pancreatic or hepatic lesions could be detected by PET/CT using established tumor markers such as [^11^C]HTP (B, E, H) and [^18^F]FDG (C, F, I). Reproduced from 26.

The development and clinical introduction of ^68^Ga is accelerating 117 and ^68^Ga has been employed to label several exendin-4 analogues. The uptake of the radiopharmaceuticals could be localized with high contrast in pancreas and insulinoma lesions. A case examination of a patient with severe hypoglycemia was conducted using an [^68^Ga]Ga-DO3A-VS-Cys^40^-Exendin-4 PET/CT 26. Multiple small liver metastases and paraaortal lymph node lesions were clearly visualized (Figure 5), while computed tomography, ultrasound, [^18^F]Fluorodeoxyglucodse/PET-CT or [^11^C] 5-Hydroxytryptophan/PET-CT could not provide conclusive results. [^68^Ga]Ga-DO3A-Exendin-4/PET-CT examination impacted the treatment of the patient and thus demonstrated its potential for the management of this disease 26. Clinical study where 5 patients with endogenous hyperinsulemic hypoglycemia were enrolled was conducted 87. [Nle^14^,Lys^40^(Ahx-DOTA-^111^In)NH_2_]exendin-4 and [Nle^14^,Lys^40^(Ahx-DOTA-^68^Ga)NH_2_]exendin-4 87 were compared in terms of detection rate, resolution, and background uptake. [Nle^14^,Lys^40^(Ahx-DOTA-^68^Ga)NH_2_]exendin-4 correctly identified the insulinoma in 4 of 4 patients, whereas [Nle^14^,Lys^40^(Ahx-DOTA-^111^In)NH_2_]exendin-4 SPECT/CT correctly identified the insulinoma in 2 of 4 patients. [Nle^14^,Lys^40^(Ahx-DOTA-^68^Ga)NH_2_]exendin-4 was shown to be sensitive in localizing hidden benign insulinomas and was found superior in terms of shorter examination time, higher tumor-to-background ratio, higher spatial resolution, lower radiation dose, and accurate quantification.

**Figure 6:**
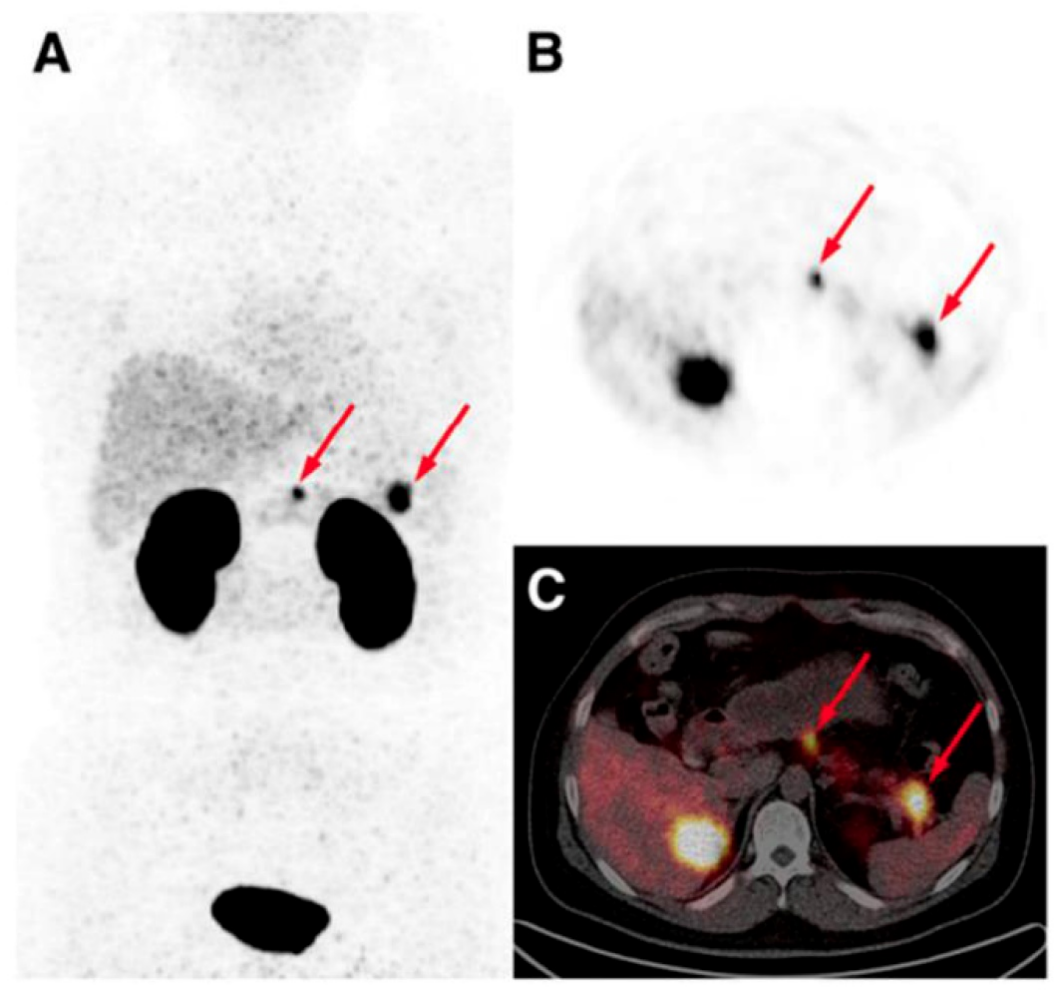
Maximum intensity projection (A) and axial PET (B) and PET/CT (C) images obtained from a patient 40 min post administration of ^68^Ga-NOTA-exendin-4. Arrows point at two lesions in neck and tail of pancreas that were surgically removed and confirmed to be insulinomas histologically. Reproduced from 38.

The detection of occult insulinoma was enabled by ^68^Ga-NOTA-MAL-Cys^40^-exendin-4 PET/CT and subsequent surgical removal of the pancreas tail insulinoma resulted in recovery from hypoglycemia 75. The imaging was performed 2 h post injection in order to decrease the kidney uptake and allow visualization of the pancreas tail. In the subsequent prospective study, the authors explored the potential of ^68^Ga-NOTA-exendin-4 PET/CT for the detection of insulinomas in a larger patient cohort and found sensitivity in the localizing of the lesions of 97.7% which was considerably higher than that of CT (74.4%), MRI (56.0%), EUS (84.0%), and ^99m^Tc-HYNIC-TOC (19.5%) (Figure 6) 38. Lesions as small as less than 1.0 cm were detected by ^68^Ga-NOTA-MAL-Cys^40^-exendin-4 PET/CT in 11 patients. The kidney uptake was high interfering with the detection of pancreas tail lesions, however additional examination 2-3 h post injection resulted in unambiguous delineation. Noteworthy, the only patient diagnosed with malignant insulinoma showed high uptake in both ^68^Ga-NOTA-MAL-Cys^40^-exendin-4 PET/CT and ^99m^Tc-HYNIC-TOC. [Lys^40^-(Ahx-DOTA-^68^Ga)NH_2_] PET/CT clearly delineated pancreatic lesion while [Lys^40^-(Ahx-DOTA-^111^In)NH_2_] SPECT/CT was not conclusive in an intrapatient comparative study 76. The high GLP-1R expression was confirmed on the tissue after the pancreatectomy that resolved the hypoglycemia. The density of GLP-1R was 3-fold higher in the islets of this nesidioblastosis patient as compared to that of normal pancreas islets implying that [Lys^40^-(Ahx-DOTA-^68^Ga)NH_2_] PET/CT may be a valuable tool in determining the surgical strategy also in nesidioblastosis which can be a focal disease. A lesion located at the proximal jejunum, below the body of pancreas and multiple liver metastases were clearly detected by ^68^Ga-exendin-4 PET/CT enabling efficient treatment of the patient 152. ^68^Ga-DOTA-exendin PET/CT aided conclusive diagnosis accurately localizing the culprit lesion for the subsequent surgery 153. The patient experienced complete postoperative recovery. ^68^Ga-DOTA-exendin PET/CT was the only method that could visualize the pancreatic lesion and thus facilitate curative surgical treatment 154, 155. Detection of an insulinoma lesion using ^68^Ga-DOTA-exendin PET/CT enabled subsequent ultrasound-guided ethanol ablation and monitoring of the tumor response to the treatment 156. A large multicenter study comparing ^68^Ga-NODAGA-exendin-4 with ^68^Ga-DOTATATE in 56 subjects with adult hyperinsulimemic hypoglycemia is ongoing (ClinicalTrials.gov identifier: NCT03189953) and is expected to clarify the role of ^68^Ga labeled exendin-4 in the management of this group of diseases including insulinoma.

GLP-1R imaging has potential in metabolic disease in human given its expression in human pancreatic islets and the incretin effect. Thus, there are several clinical trials ongoing that aim to elucidate the impact of ^68^Ga-exendin-4 PET imaging in different aspects of metabolic disease. Hitherto, clinical results on ^68^Ga-exendin-4 PET have not yet been published in journal format, thus this is an overview of studies published in international trial database (clinicaltrial.gov). The GLP-1R expression in pancreas (assumed to be correlated to the beta cell mass) is investigated by ^68^Ga-NODAGA-exendin-4 PET/CT in T1D subjects with unstable and stable glycaemic control (NCT03785275) as well as during the honeymoon phase in T1D (NCT03917238) and in subject with gestational diabetes (NCT03182296). Furthermore, the possibility to detect functional islets in the liver of T1D subjects with intraportally transplanted islets is also evaluated (NCT03785236).

GLP-1R expression is also evaluated in subjects with T2D undergoing gastric bypass (NCT02542059). Finally, the value of ^68^Ga-NODAGA-exendin-4 in management of congenital hyperinsulinism in comparison with^ 18^F-DOPA and contrast enhanced CT (NCT03768518) is under investigation. The results of the abovementioned studies are expected to improve the understating of GLP-1R expression in health and disease, as well as the notion of using ^68^Ga-exendin4 as a marker for beta cell mass.

### Side effects

In a preclinical PET imaging study with [^68^Ga]Ga-DO3A-VS-Cys^40^-exendin-4, pigs developed tachycardia after intravenous administration 94, 96, however to the best of our knowledge the published patient studies did not present such adverse effects. In some patients, transient palpitation at the time of injection that lasted a few seconds has been reported 38. Slight plasma glucose concentration reduction but no severe hypoglycemic episodes were observed in a study of 6 patients with endogenous hyperinsulinemic hypoglycemia 66. One patient experienced a short episode of vomiting, which may be expected at high doses of GLP-1 agonist being known to affect appetite and nausea. Moreover, clinical studies on the treatment of T2D patients with exenatide (synthetic exendin-4) over twelve weeks did not demonstrate clinically meaningful effects on heart rate 157 even though GLP-1R is expressed in heart 158.

### Intraoperative application

Another application elevating success of a surgery is intraoperative use of gamma-probe detecting ^111^In-DOTA-exendin-4 accumulated in the lesions 66, 159. The method offers crucial advantage over intraoperative venous sampling which is a complex procedure with potential complications 36. Fluorescent exendin probes could similarly accumulate in insulinoma lesions, and potentially offer improved resolution and delineation of tumors during removal compared to long-lived SPECT radionuclides. The combination of radionuclide and fluorescently labeled GLP-1R targeting probes potentially offers advantage of first localizing the tumor by whole body PET/SPECT imaging, followed by using the fluorescent signal for guiding accurate tumor removal 101.

## Theranostics/Radiotheranostics in GLP-1R targeting

Non-invasive imaging targeting GLP-1R can be used for the selection of treatment, monitoring treatment response, dose planning for the treatment based on both radioactive (radiotheranostics) and non-radioactive (theranostics) pharmaceuticals 160. It is a promising tool in both diabetes and cancer.

### Theranostics in diabetes

GLP-1 analogues are of strong interest in the context of theranostics since the treatment of the diabetes is also targeted at GLP-1R (the broad class of GLP-1 agonists) and thus the drug efficacy and dose can potentially be predicted and planed individually and the patient response to the drug can be monitored enabling adjustment of the treatment respectively. Little is known of the numerical variation of GLP-1R expression in the human pancreas, but initial data from imaging studies with radiolabeled exendin-4 indicates significant variation in individuals. This observation combined with the notion that some patients develop tolerance to GLP-1 agonists indicate a potential area of application for exendin-4 based imaging.

Quantitative GLP-1R imaging has been proposed for the assessment of drug mediated occupancy in the pancreas both preclinically and clinically. This could theoretically be used to benchmark different GLP-1 agonists versus each other, as well as assist in improving understanding of the dose-effect relationship. This is particularly important for GLP-1R agonist since there is likely an optimal dosing and exposure interval with adequate clinical efficiency, while doses exceeding this may induce side effects, e.g. nausea.

The in vivo monitoring and quantification of the endogenous and transplanted beta cells would provide crucial information on the cell survival and the loss of functionality. Prospective in vivo studies for the measurement of beta cell mass in diabetic patients and healthy individuals would potentially allow understanding of underlying disease mechanisms and assignment of individualized treatments. Stratification of patients depending on levels of functional beta cells in pancreas may even impact the diagnosis, as the current major classifications of diabetes (T1D, T2D, gestational diabetes) may be too simplified and do not accurately describe the underlying disease progression 161, 162. Novel sub-categories of diabetes have been proposed based on phenotypical and metabolic characteristics. Furthermore, it is expected that novel imaging techniques for BCM quantification could contribute to such phenotypic characterization in the future.

The abovementioned predictive theranostic applications require precise, non-invasive, quantitative method that would allow repetitive examinations as potentially subtle changes or differences in GLP-1R expression or BCM must be detected and quantified. Broader deployment of exendin-4 mediated GLP-1R PET scanning in the clinic for theranostic studies (outside of insulinoma management) as described above is based on the availability of some crucial data. Some of the data is expected to be available based on the outcome of already ongoing clinical trials. Current preclinical data clearly support the notion that the GLP-1R expression can be quantified in the pancreas with high precision by PET, but this precision must be verified in clinical PET studies (see for example ongoing study NCT03350191). Additionally, ^68^Ga-exendin-4 PET outcome is currently usually reported as SUV uptake in pancreas at certain time points (often some interval between 50-90 minutes after administration). This semi-quantitative assessment has never been benchmarked against a full kinetic model including arterial input corrected for metabolic stability of the radioligand. Such a comparison would reduce the ambiguity of the semi-quantitative endpoint and instead objectively point to a time-point and scanning duration were the SUV measurement correlates with the golden standard PET model assessment. Furthermore, such validation would remove the future need of including venous or arterial sampling (i.e. reduce the invasiveness of the procedure) as well as replacing the need for dynamic scanning with a static pancreatic scan of shorter duration (i.e. improve patient comfort and examination throughput). Again, also these outstanding questions may be answered by the outcome of NCT03350191. Finally, the notion of ^68^Ga-exendin-4 as a surrogate marker for human pancreatic BCM is dependent on the outcome of study NCT03889496 that will verify the important islet-to-exocrine binding ratio of radiolabeled exendin-4 by post resection autoradiography of the pancreas in human.

The long-term outcome of several clinical studies on intraportal islet transplantation, including the Edmonton protocol 8 have demonstrated that monitoring of transplanted islets in vivo is crucial for assessing the efficacy of different transplantation procedures. PET imaging of prelabeled islets demonstrated significant islet loss during the acute peri-transplant phase 163, but longitudinal repeatable and direct assessment of engrafted viable islet mass are lacking in the clinic. Healthy islets were isolated from the pancreas of a patient that underwent insulinoma surgery and were re-implanted into brachioradialis muscle 10. The beta cells could successfully be visualized in vivo using [Lys^40^(Ahx-DTPA-^111^In)NH_2_]exendin-4 one year after the transplantation. High interindividual variation was observed in patients affected by T1D 57 indicating that the individual approach to the treatment regimen is required to monitor response and adjust the dose or alter the medication for the improved treatment outcome. Moreover, a substantial variation in beta cell mass is found also amongst healthy subjects 55.

### Radiotheranostics in cancer

Radiotheranostics for the management of neuroendocrine patients is the most prominent and pioneer example. The radionuclide ^68^Ga and ^177^Lu pair is the most frequently used one in the context of PRRT. Both Ga(III) and Lu(III) form stable complexes with DOTA chelator conjugated to somatostatin analogues, in most cases allowing for the use of the same ligand molecule to assure the least variation in the biodistribution pattern. The investigation of the feasibility of the similar approach for ligands targeting GLP-1, CCK, and GIP receptors is of utmost interest 164. The radiotherapeutic effect of [Lys^40^(Ahx-DTPA-^111^In)NH_2_]-exendin-4 was demonstrated in mice with Rip1Tag2 spontaneous insulinoma 88. The tumor reduction was observed in radioactivity dose dependent manner by up to 94%. The therapeutic effect was assigned to tumor cell apoptosis, necrosis, and decreased proliferation.

#### Dosimetry and feasibility of radiotheranostics

Pre-therapeutic imaging in the context of internal targeted radiotherapy has two major objectives: 1. Dosimetry investigation wherein radiation absorbed dose to healthy organs is measured to assess the potential radiotoxicity; 2. Estimation and planning of radiation dose that would provide effective radiotherapy on one hand and safe radiation dose to the healthy organs on the other hand. Dosimetry investigation plays a crucial role in the radiopharmaceutical development in terms of estimating the number of annual examinations that can be conducted without presenting hazard to normal organs as well as in the context of internal radiotheranostics. The major factors that influence the radiation dose is the biodistribution, excretion, and radionuclide decay characteristics such as half-life and radiation type.

High kidney uptake with subsequent high radiation dose is the major factor precluding the radiotherapeutical use of exendin-based analogues labeled, e.g. with ^111^In and ^177^Lu 26, 59, 66, 75, 88. For example, [Lys^40^(Ahx-DTPA-^111^In)NH_2_]-exendin-4 accumulated in kidneys with high absorbed radiation dose caused morphological changes thus hindering radiotherapeutic application of ^111^In. However, it should be mentioned that ^177^Lu is more efficient than ^111^In as experience with somatostatin radiotherapy indicates. Lower kidney uptake observed for the iodine-based analogues would present an advantage from the radiotheranostic point of view with positron emitting ^124^I and radiotherapeutic ^131^I 160. However, initial results of such analogues demonstrated fast washout of ^125^I-BH-Ex(9-39)NH_2_ radioactivity from mouse xenograft 128 and therapeutic outcome using respective ^131^I labeled analog has not been demonstrated. Additional advantage of using ^123,124^I/^131^I imaging/radiotherapeutic pair would be the identical chemical structure of the radiopharmaceuticals providing identical biodistribution pattern.

The physiological expression of GPL-1R in the endocrine pancreas, intestine, lung, kidney, breast and brain potentially may cause difficulty due to interference of the respective background uptake with the target uptake and radiation dose to those healthy organs. However, it should be mentioned that the clearance from blood and healthy organs without GLP-1R expression for the majority of exendin analogues is fast, and it does not impose dosimetry issues with ^68^Ga, ^99m^Tc, or ^177^Lu 59, 86. This is a very crucial factor with respect to radiation sensitive organ such as red marrow. Other organs with relevant expression of GLP-1R could conceivably exhibit significant binding of radiolabeled exendin and in turn deposit a high absorbed dose in surrounding tissues. However, the dosimetry in lung for example, with relevant physiological GLP-1R expression, has been shown to pose no concerns with regards to radiation safety 26, 58, 70. Moreover, human lung express GLP-1R to lesser extent 32 as compared to rodents that the preclinical experiments were conducted with. The uptake of radiolabeled exendin analogues in the lung can be attributed not only to the presence of GLP-1R 68, but also megalin receptors as it was demonstrated in megalin-deficient mice 135.

Lower radiation burden from ^68^Ga-labeled analogues as compared to ^64^Cu 78 and ^111^In 69 labeled counterparts was demonstrated pre-clinically. Effective dose for [Lys^40^(Ahx-NODAGA-^68^Ga)NH_2_, Nle^14^]-exendin-4 was 12-fold lower than that for [Lys^40^(Ahx-NODAGA-^64^Cu)NH_2_, Nle^14^]-exendin-4 78. A comparative study of [Lys^40^(Ahx-DOTA-^111^In)NH_2_]-exendin-4, [Lys^40^(Ahx-DOTA-^68^Ga)NH_2_]-exendin-4, and [Lys^40^(Ahx-hydrazinonicotinamide [HYNIC]-^99m^Tc)NH_2_]-exendin-4 conducted in Rip1Tag2 mouse model of pancreatic beta cell carcinogenesis showed the highest effective radiation dose from ^111^In-labeled analogue (155 µSv/MBq) followed by ^68^Ga- (31.7 µSv/MBq) and ^99m^Tc-labeled (3.7 µSv/MBq) counterparts 69. Comparison of various exendin-4 based imaging analogues reveals the lowest kidney dose and effective dose for ^68^Ga 95 followed by ^18^F 108, ^99m^Tc 69, ^64^Cu 78, and ^111^In 69.

**Figure 7:**
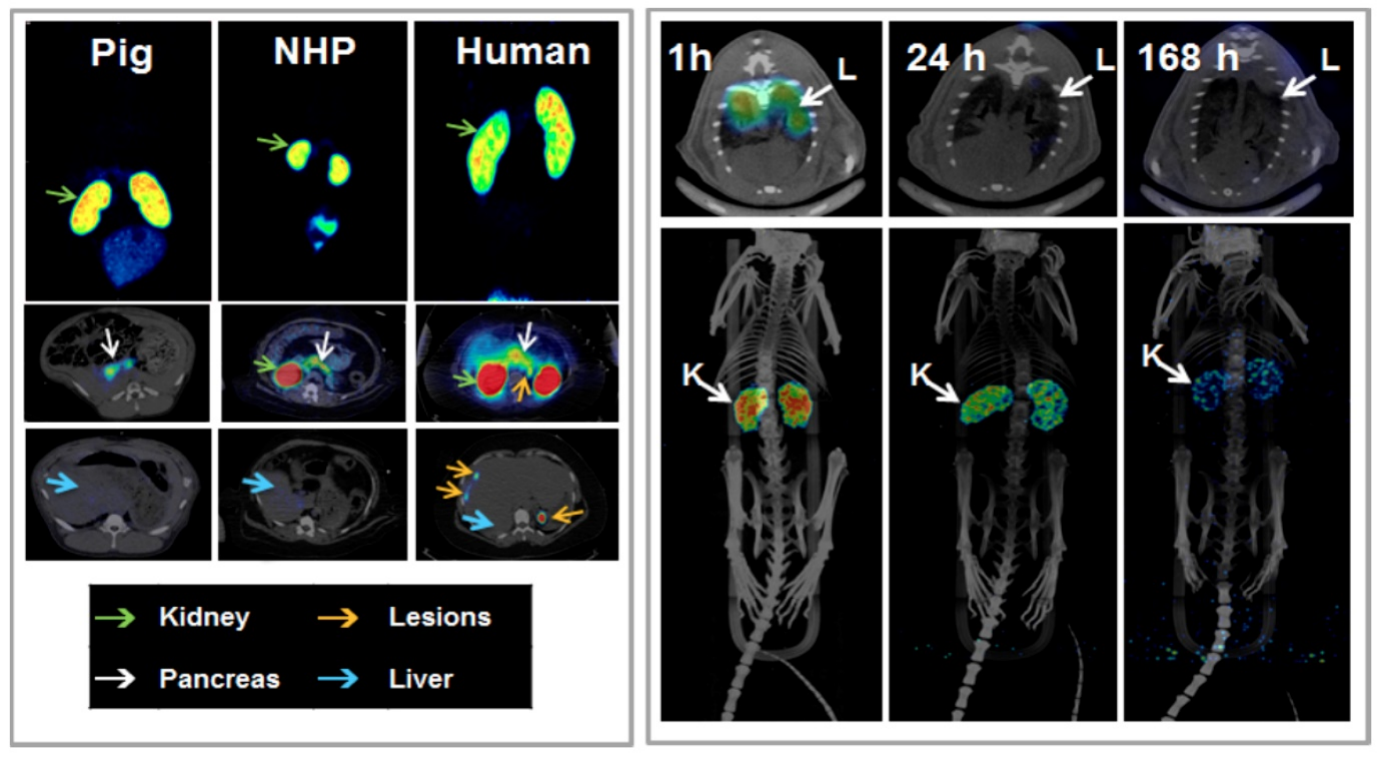
** Left panel:** *In vivo* biodistribution of [^68^Ga]Ga-DO3A-VS-Cys^40^-exendin-4 as analyzed by PET-CT imaging in the pig (0.025 µg/kg; 60 mi), non-human primate (NHP) (0.01µg/kg; 90 min), and human (0.17 µg/kg; 40 min, 100 min and 120 min). The pancreas (white arrow) was delineated within 10 minutes post injection in all species. The low hepatic uptake (blue arrow) shows the potential for outlining insulinoma tumor metastasis (orange arrow, human images). The MIP coronal images demonstrate the highest uptake of the tracer in the kidneys (green arrow) in all species. **Right panel:** Representative fused SPECT-CT images of [^177^Lu]-DO3A-VS-Cys^40^-exendin-4 in rats at different time points. Lungs could be outlined at 1 h p.i. and showed faster clearance in later time points (upper panel). MIP images of whole-body scan showing dominance of kidneys as excretory organ of tracer (lower panel). Reproduced from 115.

[^68^Ga]Ga-DO3A-exendin-4/PET-CT demonstrated clinical value for the diagnostic imaging and guided surgery of insulinoma patients 26. The subsequent receptor targeted internal radiotherapy using ^111^Lu-labeled analogue would be of considerable benefit for the treatment, however a thorough investigation of dosimetry for both [^68^Ga]Ga-DO3A-exendin-4 and [^177^Lu]Lu-DO3A-exendin-4 in rat, pig, non-human-primate and a human showed high kidney absorbed dose (Figure 7) 26, 58, 59, 94, 95.

The human extrapolated dosimetry for [^68^Ga]Ga-DO3A-VS-Cys^40^-exendin-4, predicted from rat, pig or NHP was favorable and potentially would allow for repeated imaging in individuals before reaching the limiting absorbed dose either in the critical organ (kidney) or the effective whole-body dose 95. Several examinations annually would be possible allowing longitudinal studies using [^68^Ga]Ga-DO3A-exendin-4 and treatment response monitoring. The human predicted dosimetry for [^177^Lu]Lu-DO3A-VS-Cys^40^-exendin-4 assessed from rat biodistribution also identified the kidney as the critical organ 59. It was estimated that only approximately 4 GBq of [^177^Lu]Lu-DO3A-VS-Cys^40^-exendin-4 could be administered before reaching the maximal tolerated kidney dose (23 Gy) meaning that a therapeutically meaningful dose of [^177^Lu]Lu-DO3A-exendin-4 could cause irreversible damage to kidneys. Thus, the use of renal protective agents, or other means of reduced kidney uptake is likely required before considering [^177^Lu]Lu-DO3A-VS-Cys^40^-exendin-4 for insulinoma radiotherapy. Interestingly, despite the specific accumulation in the pancreatic cells, no acute diabetogenic effects in rats could be observed during a week 59.

The improvement of in vivo stability of exendin-based radiopharmaceuticals, as mentioned above, not only would possibly decrease the kidney uptake, but also improve the lesion uptake delivering higher radiation dose. The neutral endopeptidase (NEP) presumably causes 50% degradation of the GLP-1 ligand in blood circulation, and the inhibition of NEP improves the ligand stability 145. The higher stability might result in the redirection of the radiopharmaceutical to the GLP-1R expressing lesions.

## Conclusions

There are unmet medical needs in both diabetes and oncology that might be met by molecular imaging and therapy providing target specific individualized approach. Considerable progress both in radiopharmaceutical and technological development has been made during last two decades. Radiopharmaceuticals based on most commonly used metal and halogen radionuclides were developed offering various advantages. Imaging diagnostics using exendin based analogues targeted at GLP-1R in combination with SPECT and PET has proven its clinical value in insulinoma management, while many potential clinical uses in metabolic diseases including diabetes and islets transplantation are under investigation. Internal targeted radiotherapeutic application in oncology is remaining unrealized due to the unfavorable distribution and high radiation absorbed dose to kidneys. The research to reduce kidney and enhance tumor uptake continues and shows novel approaches and progress.

## Figures and Tables

**Table 1:**
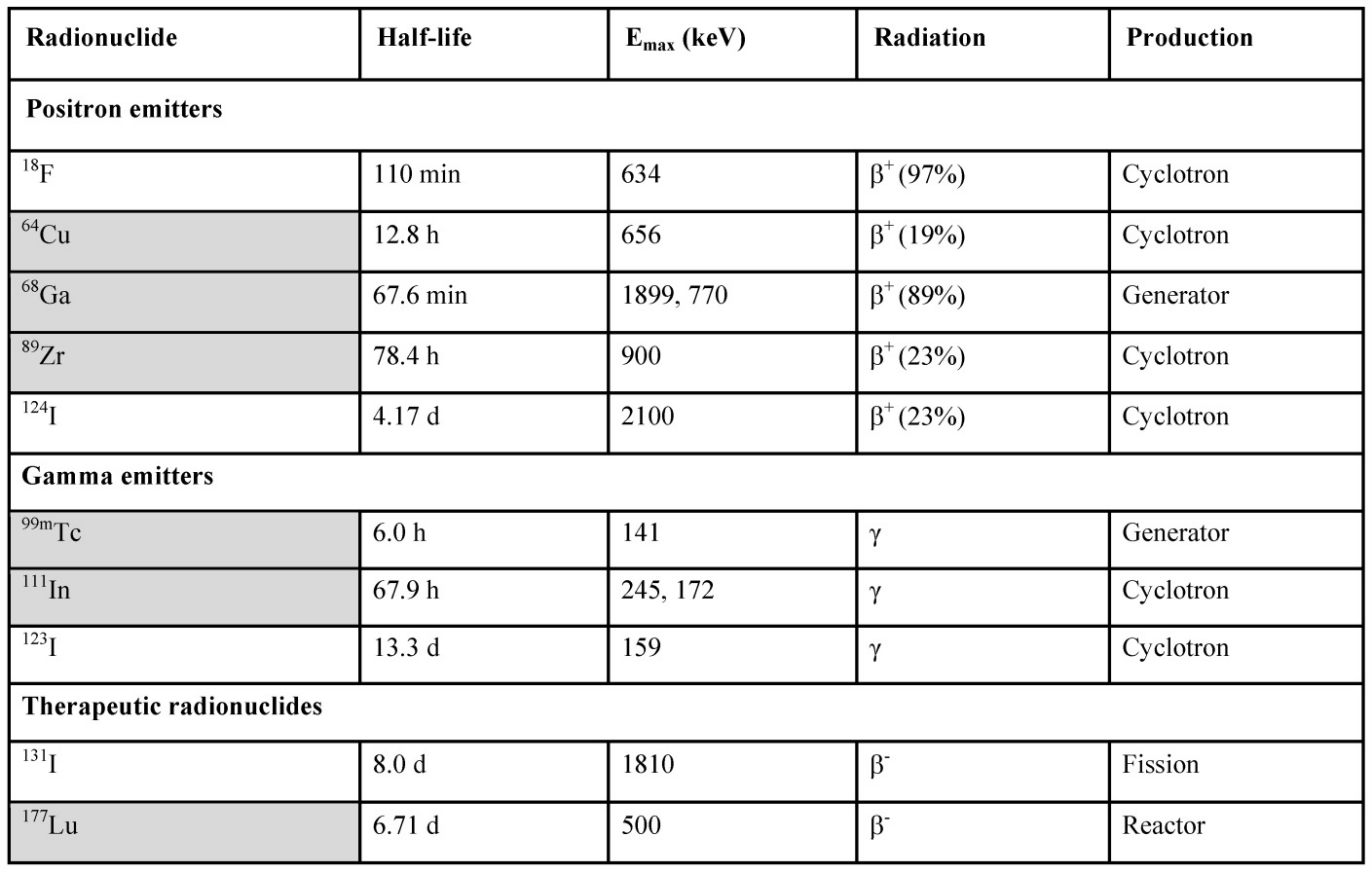
Radionuclides used for the labeling of exendin analogues in respective fields of PET, SPECT and radiotherapy, their production mode and decay properties.

Radiometals are marked in grey.
